# Modulating properties of solid carbon nanospheres *via* ion implantation with hetero-ions

**DOI:** 10.1039/d5na00616c

**Published:** 2025-08-28

**Authors:** Joyce B. Matsoso, Kamalakannan Ranganathan, Daniel Wamwangi, Rudolph Erasmus, Neil J. Coville, Trevor Derry

**Affiliations:** a Molecular Sciences Institute, School of Chemistry, University of the Witwatersrand Johannesburg 2050 South Africa neil.coville@wits.ac.za; b DSI-NRF Centre of Excellence in Strong Materials, University of the Witwatersrand Johannesburg 2050 South Africa trevor.derry@wits.ac.za; c Materials Physics Research Institute, School of Physics, University of the Witwatersrand Johannesburg 2050 South Africa

## Abstract

Solid carbon nanospheres of ∼200 nm diameter have been prepared and then doped by ion implantation, using a specialized end-station adapted for the uniform implantation of powders. Boron, nitrogen and neon ions were chosen, the latter for control purposes. Herein, the dependence of the physicochemical properties of solid carbon spheres on the fluence of the implanted ions was investigated by controlling the addition of the 100 keV of B^+^, N^+^ or Ne^+^ ions into the carbon shell over 7 h and 14 h implantation periods at room temperature. SEM analysis revealed significant surface deformation in the form of cracks for the Ne^+^ implanted samples, whilst little structural deformation was observed with N^+^ and B^+^ implanted samples. Furthermore, TEM micrographs confirmed the dependence of the structural properties on the ion fluence. Finally, magnetic properties showed that the type of the hetero-ion as well as the affiliation of the carbon with the bonding configurations of the hetero-ion influenced the transition from diamagnetism to super-paramagnetism as absolute zero was approached. The Néel temperature varied somewhat but was below about 10 K. Boron conferred a much greater paramagnetic susceptibility at low temperature than the other ions and showed indications of a higher electrical conductivity at higher temperatures, suggesting an electronic doping effect. The study highlights the impact of the choice of the heteroatom ion on the properties of the solid carbon spheres for the development of next generation carbon-based electronic devices.

## Abstract 2

Dopant fluence on the physicochemical and conductivity properties of carbon spheres.

## Graphical abstract

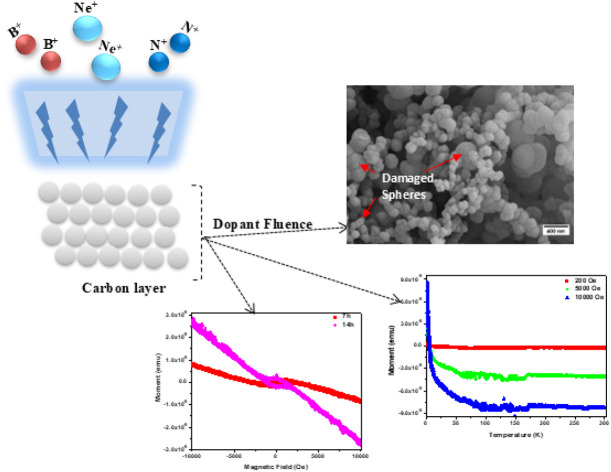

## 1. Introduction

Carbon materials have garnered tremendous attention within the nanotechnology world ever since the discovery of fullerenes in the mid-1980s.^1^ Much of the upsurge of interest in carbon materials is driven by the ease of formation of various structures and shapes, especially in the ‘nano’ regime.^2,3^ Such shaped carbon nanostructures include materials like tubes, fibres, spheres, onions, horns, and many others.^3–9^ Among the numerous forms of the structured carbon materials, carbon spheres enjoy a long history of study and exploration in various technological fields due to their unprecedented properties.^10–14^ Typically, the generic term carbon spheres is commonly used to refer to spherical or near spherical structured carbon-based materials, and depending on the synthesis route, these nanostructures could exhibit a solid or hollow morphology.^12,13,15^ The properties of solid carbon spheres (SCSs) can be modified by the incorporation (doping) of heteroatoms into the carbon network of the SCSs by using a variety of techniques such as chemical vapour deposition (CVD), arc-discharge, and hydrothermal, and these processes improve their range of application in nano-scale electronic devices.^16–19^ Naturally, the electron acceptor boron (B) and electron donor nitrogen (N) are ideal candidates for easy incorporation into the carbon network, due to their roughly similar atomic sizes to that of carbon.

Although the commonly used SCSs doping techniques may open an avenue of new and/or improved properties of the SCSs, the majority of such techniques are mostly limited to the incorporation of non-metallic heteroatoms; likely given by the high affinity of C to atoms such as B, phosphorus, sulphur and/or N atoms through formation of stable covalent bonds.^16,20–22^ The possibility of doping with very low heteroatom concentration, for fabrication of next generation microelectronics, constitutes one of the major holdups faced by most doping techniques. Furthermore, most doping techniques yield carbon materials with the dopant placed on the edge/periphery of the sphere rather than inserted inside the carbon.^16,21,22^ In order to achieve a better control of dopants in the carbon network, ion implantation with energetic particles, such as neon (Ne), argon (Ar), xenon (Xe), nitrogen (N) or boron (B), has been proven to be an efficient route for enhancing the electronic properties of carbon nanostructures, as shown by studies on nanodiamonds, carbon nanotubes, and graphene.^23,24^ In this exploratory work, we selected B and N for implantation, due to their masses and valency that are similar to that of carbon, whilst we used Ne as an inert control, with a mass closest to B and C. Most importantly, the ion-implantation technique offers an ability to fine-tune dopant addition through the control of the kinetic energy or fluence of the incident dopant ions, and to facilitate the curing of the implantation-damaged host surface *via* post-implantation annealing. These effects could allow for the fine tuning of the optoelectronic and magnetic properties of the final product. Despite the extensive research performed by ion-implantation of carbon nanostructures, to the best of our knowledge, this technique has not yet been used to modify the behaviour of solid carbon spheres. The current study was undertaken to investigate the influence of fluence on the final properties of SCSs after ion irradiation. We also hoped to evaluate the effect of the type of the dopant ion on the final properties of the SCSs. Similar measurements^25^ have been made on SCS samples, but without any addition of ion implantation.

## 2. Experimental methods

### 2.1 Synthesis of solid carbon spheres (SCSs)

A vertical chemical vapour deposition (CVD) reactor consisting of a hollow tubular quartz reactor (100 cm × 4 cm) loaded in a furnace was set up as shown in Fig. S1. Acetylene (C_2_H_2_, ≥99.995%, impurities <50 ppm, Afrox SA) and argon (Ar, ≥99.999%, impurities <10 ppm, Afrox SA) were used as the carbon source as well as both the ambient media and carrier gas, respectively. In a typical synthesis experiment, the vertical CVD furnace was fast heated to 900 °C under 100 sccm (standard cubic centimetres per minute) of Ar gas. After reaching the designated temperature, the Ar gas was completely replaced by C_2_H_2_ (200 sccm) for 15 min (ref. 2 and 26) to synthesize solid carbon nanospheres. After cooling the reactor to room temperature under a continuous flow of 100 sccm of Ar, the carbonaceous materials were purified using a Soxhlet extraction apparatus (Fig. S2), in order to remove the soluble impurities that formed. Typically, the as-synthesized carbonaceous materials were placed in a thimble and purification was carried out at reflux for 24 h using toluene solvent (Sigma Aldrich, 99.5%).^26,27^ The purification step was indicated by the colour change of toluene from colourless to a greenish-brown colour; an indication of the removal of polyaromatic hydrocarbons. The purified product, labelled as pristine solid carbon spheres (SCS_p_), was then dried at 100 °C for 12 h.

### 2.2 Ion implantation of the SCSs

Ion implantation is a materials engineering process by which ions of a certain element, such as carbon (C) nitrogen (N) or boron (B) are accelerated in an electric field and impacted onto a solid.^28,29^ An ion implant process is defined by the fluence/dose and energy of the ions. Fluence is the amount of dopant being implanted (per unit area), whereas the energy determines the effective depth of the dopant into the substance that is being doped. For the current work, a typical acceleration energy of 100 keV and a beam current of ∼100 μA were chosen for the implantation process. In order to implant one gram aliquots of the powdered SCSs samples with B, N and neon (Ne), a specialized end-station at iThemba LABS on the University of the Witwatersrand campus was used.^30^ The set-up enabled tumbling of the powder SCS materials to allow access of all particles to the dopant and thus obtain homogeneous doping in the beam. Implantations were carried out at room temperature. The samples were then labelled as SCS-B^+^, SCS-N^+^, and SCS-Ne^+^ for samples implanted with boron, nitrogen and neon ions, respectively. To evaluate the effect of the dopant fluence on the electrochemical properties of the SCSs, two implantation times of 7 h and 14 h, were used. These long implant times accord with the finely divided nature of the particles.

Simulated implant profiles (not shown here for brevity; nonetheless the profiles indicate classical behaviour) using the well-known SRIM code^31^ show that the penetration of 100 keV ions in graphitic carbon is up to 270, 230 and 200 nm for ^11^B, ^14^N and ^20^Ne respectively. Thus, most of the ions will be stopped deep inside in the particles (which are tumbled so that all surfaces are irradiated). Any ions that penetrate the carbon will be stopped elsewhere in the curtain of falling particles. After a short time, implantation becomes uniform throughout the sample. Rough calculations give the number of added ions (or atoms) as about 300 ppm or 600 ppm after 7 h or 14 h of irradiation, respectively. These estimates are based on the sum of the ions implanted (from the integrated charge), divided by the number of C atoms in 1 g of powder, giving an atomic concentration, similar to what is often achieved in implantation doping, assuming uniformity. The convention of quoting implants in terms of ions per square centimetre is inappropriate here, as the material is not a flat surface.

### 2.3 Characterization

Transmission electron microscopy (TEM) micrographs were collected on a Tecnai Spirit T12 at 120 kV acceleration voltage to determine the low and high magnification morphological features of the pristine and ion implanted SCS samples. Further morphological features were determined using the MEB Zeiss Merlin Compact scanning electron microscopy (SEM), at an accelerating voltage of 10 kV, after the samples were dispersed in ethanol, dropped on an aluminium stub and coated with a layer (10 nm) of gold–palladium. The Horiba LabRam HR micro-Raman spectrometer equipped with a laser excitation wavelength of 514.5 nm and a liquid N_2_ cooled charge coupled device detector was used for evaluation of the degree of graphitization and the structural disorder created in the carbon spheres, prior to, and after ion implantation with the heteroatoms. The textural properties were determined from the ultra-pure N_2_ adsorption and desorption isotherms using a Micromeritics Tristar 3000 instrument at 77 K, following the degassing of samples for 4 h at 100 °C. Thermal stabilities of the pristine and ion implanted samples were monitored by thermal gravimetric analysis (TGA) conjugated with weight loss derivative outputs (DTG) using a PerkinElmer 6000 thermogravimetric analyser. The magnetic properties were determined using a Quantum Design Dynacool 12-Tesla Physical Property Measurement System (PPMS) in the isotherm and isofield modes.

## 3. Results and discussion

### 3.1 Morphological analysis

**Fig. 1:**
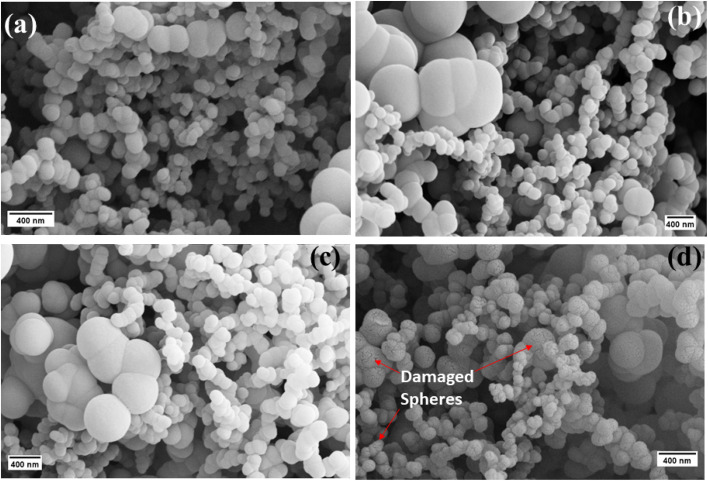
SEM micrographs of (a) pristine SCS, (b) SCS-B^+^, (c) SCS-N^+^, and (d) SCS-Ne^+^ samples after 7 h implantation time.

Surface changes of the solid carbon spheres prior to and after implantation with the different ions were investigated by scanning electron microscopy (SEM) and transmission electron microscopy (TEM) techniques. The SEM micrographs of the SCS_p_ samples (Fig. 1a) showed accreted, spherical spheres with smooth surface topography and a broad particle size distribution of about 220 ± 20 nm, with some large outliers that are typically observed in the CVD process. The latter can be attributed to the carbon source and carrier gas flow rates used during the synthesis step.^2,32,33^ Upon implantation with the heteroatom ions (^11^B, ^14^N and ^20^Ne) for 7 h (Fig. 1) and 14 h (Fig. S3) the spherical morphology of the SCSs was retained, but with possibly more agglomeration due to the effect of the deposited ion energies.

**Fig. 2:**
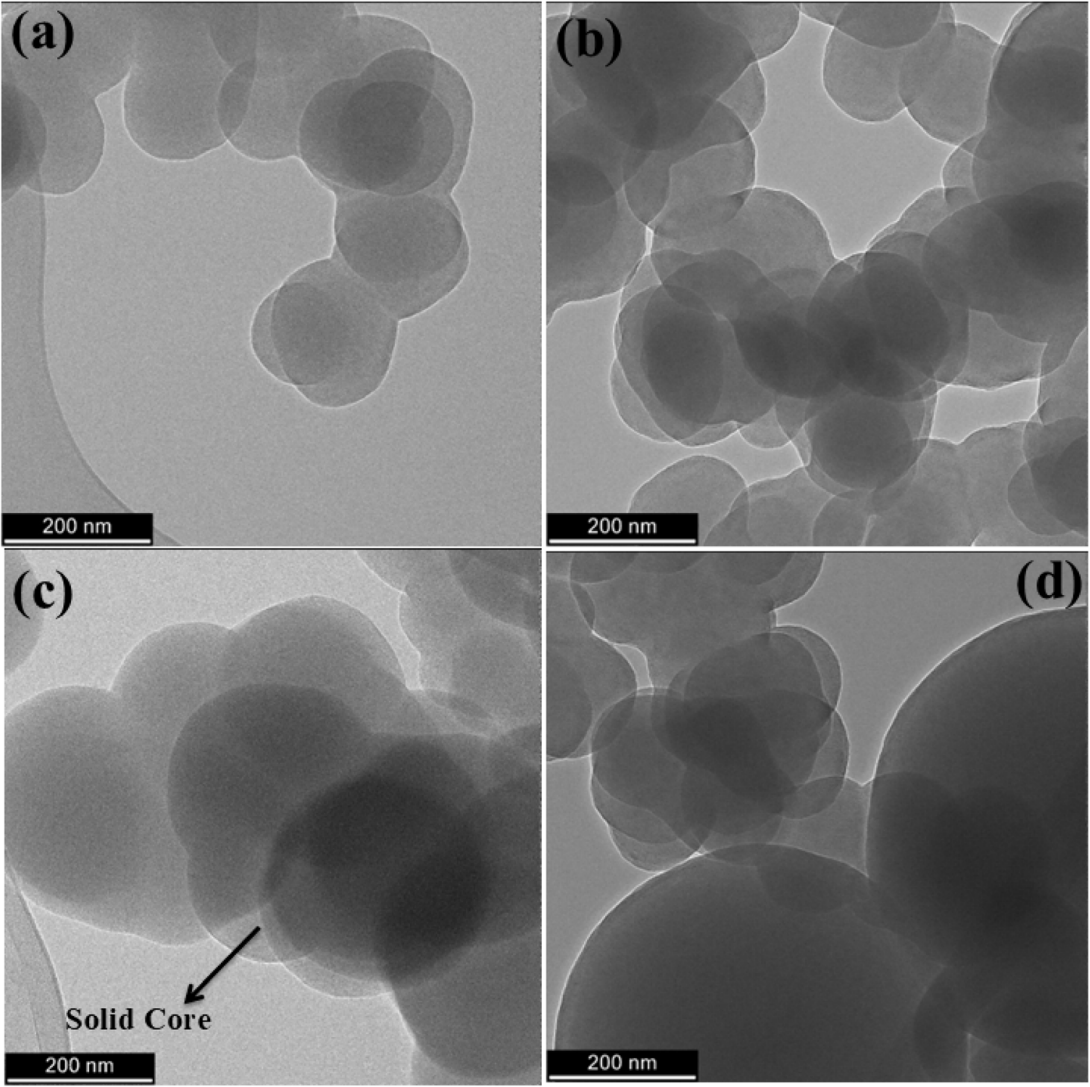
TEM images of (a) pristine SCS, (b) SCS-B^+^, (c) SCS-N^+^, and (d) SCS-Ne^+^ samples after 7 h implantation time.

Detailed changes to the structural properties of the solid spheres associated with the different ion implantation periods (7 h and 14 h) were studied from the TEM images, as shown in Fig. 2 and S4, as well as Fig. S5 and S6. The lower magnification TEM micrograph of the SCS_p_ samples (Fig. 2a) revealed an expected typical agglomerated morphology of well-defined solid nanoparticles, exhibiting an overall broad average particle size of just over 200 nm as before. Fig. S5 and S6 show the high magnification TEM images of SCS-B^+^, SCS-N^+^, and SCS-Ne^+^ samples after 7 and 14 h implantation times and indicate that little change in morphology was observed. While the particles show some heterogeneity, the effect of this on the electrical and magnetic properties was not studied here.

### 3.2 Structural studies by Raman spectroscopy

**Fig. 3:**
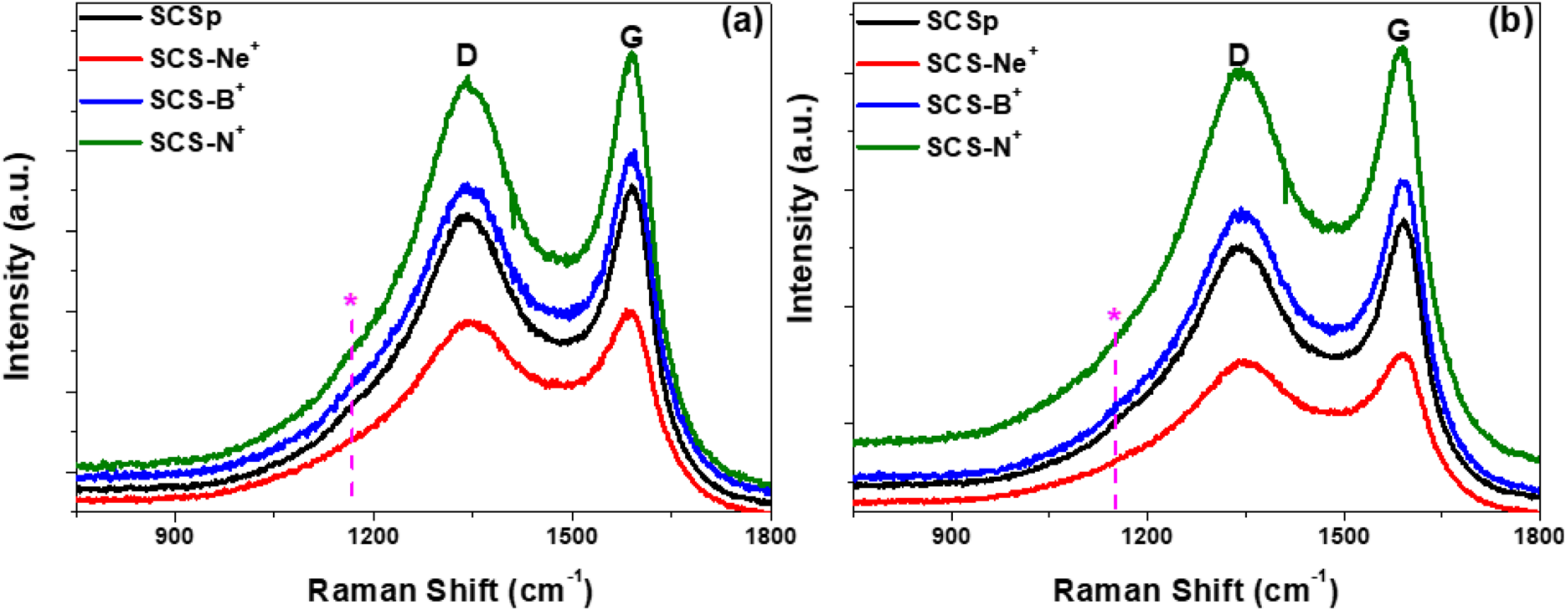
Raman spectra of the pristine SCS and ion implanted SCS samples after (a) 7 h and (b) 14 h implantation times.

Structural distortions associated with the introduction of the different ions into the solid carbon spheres were determined from the Raman spectra. As expected, the spectra (Fig. 3) showed the characteristic fingerprint first order Raman active modes for carbonaceous materials.^34,35^ The D-band, which represents the breathing modes of sp^2^ and sp^3^ carbon atoms of the six-fold aromatic rings in a carbon network, was located at ∼1337–1347 cm^−1^. On the other hand, the G-band corresponding to the E_2g_ phonon mode at the Brillouin zone centre, and indicating the presence of the graphitic sp^2^ phase, was observed at ∼1585–1590 cm^−1^. In addition to the first order active modes, a shoulder at ∼1151–1169 cm^−1^ (illustrated by the * in Fig. 3) is evidence of structural disorder of sp^3^ hybridized carbons similar to those of diamond-like amorphous carbon.^36,37^ The positions, FWHM, and intensities of all the Raman active modes were determined to permit quantification of the local structural disorder of the pristine and ion-implanted samples.

**Fig. 4:**
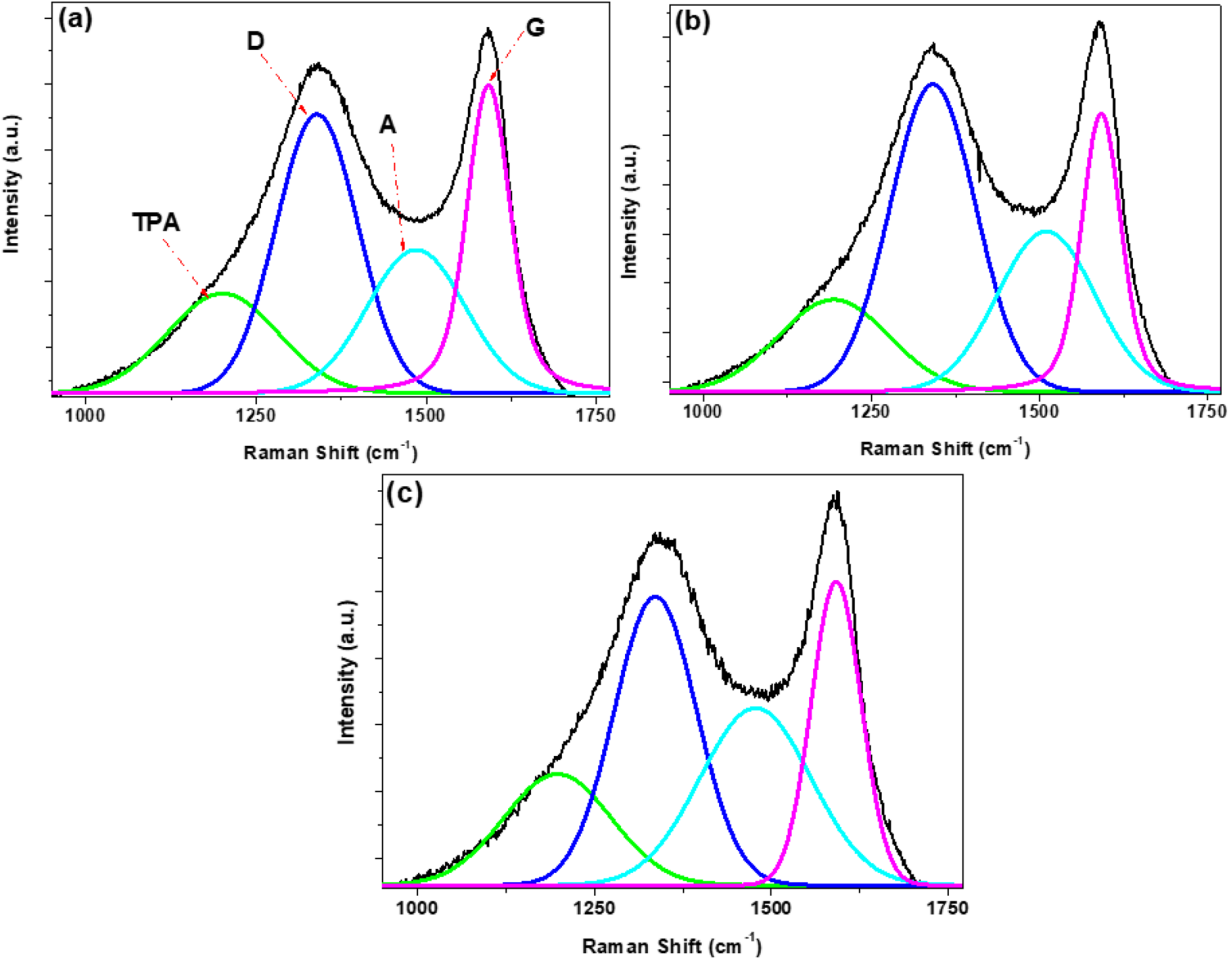
Peak fitted Raman spectra of (a) SCS-B^+^, (b) SCS-N^+^ and (c) SCS-Ne^+^ samples after 7 h implantation times.

The *trans*-polyacetylene (TPA), A-, D- and G-peaks were fitted from the experimental Raman spectra through spectral fitting of the 7 h ion-implanted samples as shown in Fig. 4. The corresponding fits for the pristine and 14 h ion-implanted samples are illustrated in Fig. S7. For all samples, the spectra were fitted to at least four component peaks located at ∼1159–1214 cm^−1^, ∼1331–1346 cm^−1^, ∼1464–1510 cm^−1^, and ∼1583–1593 cm^−1^ corresponding to the vibrational modes of the defect-induced TPA-like structures, the defect-induced D-band, and the amorphous carbon domains (A-band), as well as the sp^2^-carbon activated G-band, respectively.^38,39^ The TPA-band shows the presence of polymeric dangling chains at the zigzag edges of the carbon sphere shells, and most important, the band line width is representative of the amount of zig-zag defective edges on the shells of the carbon spheres.^35,40^ Additionally, the A-band indicates the presence of amorphous carbon, such as interstitial disordered carbon, located either on the inside or the outside planes of the shells of the carbon spheres.^41–44^ As a result, the broadness of the A-band allows an assessment of the sp^3^-carbon content, the content of Stone–Wales defects as well as that of the carbon adatoms within the curvatures of the shells of the carbon spheres.^45–47^ From Fig. 4 and S7, it was evident that the type of ion and the fluence time had different effects on the structural properties of the implanted spheres.

Therefore, given the significance of the FWHM of the defect-induced Raman active modes on quantifying the structural properties of the carbon-based materials, the influence of the implanted ions as well as the fluence time on the structural properties of the implanted SCSs samples was investigated in detail. For instance, a broad linewidth of the TPA-band was observed for the SCS-Ne^+^ samples (*ca.* ∼183 cm_7 h_^−1^ and ∼229 cm_14 h_^−1^, Fig. S8a), an indication of a large amount of zig-zag chains on the carbon sphere shells due to the implantation of heavier ions, consequently leading to the formation of a disordered carbon shell for the solid carbon spheres. Interestingly, B^+^-implanted samples exhibited narrow TPA bands (*ca.* ∼178 cm_7 h_^−1^ and ∼179 cm_14 h_^−1^, Fig. S8a) in comparison to the N^+^ ion, as evident from the broader TPA bandwidth of the N^+^-implanted samples (*ca.* ∼194 cm_7 h_^−1^ and ∼210 cm_14 h_^−1^, Fig. S8a). The difference in the formation of the edge zig-zag defective regions could be attributed to the varying affinity of ions for the carbon lattice. In particular, the broad TPA-band for the SCS-Ne^+^ suggested a weak interaction of the carbon lattice with the Ne^+^ ions, thereby prompting bonding of the ions at the edges. On the other hand, the strong interactions of carbon with both boron and nitrogen led to formation of rather stable in-plane bonds, hence the observed narrower TPA-bands. It is clear however that other factors can impact on the TPA bandwidth as the SCS-Ne^+^ was not the widest; instead, SCS-N^+^ exhibited the broadest width.

The significance of the ion mass and fluence was further reflected by the FWHM to the D- and A-bands. For instance, the strong interactions for both B^+^ and N^+^ ions led to formation of fewer sp^3^-carbon species (Fig. S8b) in interstitial sites (Fig. S8c). Interestingly, a prolonged implantation time (14 h) reduced the amount of amorphous carbonaceous impurities for both SCS-B^+^ and SCS-N^+^, thus indicating the importance of the interaction of the ion with the carbon lattice. On the other hand, the poor interaction of the Ne^+^ ions with the carbon lattice led to high lattice distortion *via* generation of adatoms, carbon radicals, and/or sp^3^ structures, resulting in more defective regions (Fig. S8b).

**Fig. 5:**
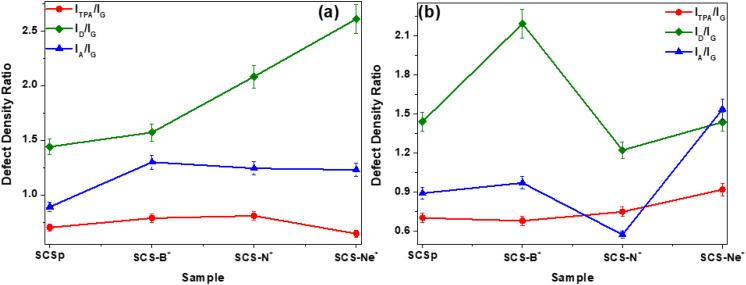
Defect density ratios of the pristine SCS and ion implanted SCS samples after (a) 7 h and (b) 14 h implantation times. The connecting lines serve only as a guide for the eye.

Finally, the degree of distortion as a function of both the type of the ion and the implantation time was investigated by determining the ratios of the integrated areas of the active defect-sensitive Raman bands (TPA-, D- and A-band) to that of the G-band,^48,49^ as shown in Fig. 5. Expectedly for all samples, the general structural defect density ratio (Fig. 5, *I*_D_/*I*_G_, green diamonds) was determined to be >1, thus denoting a low degree of graphitization at both implantation times. At 7 h implantation time (Fig. 5a), the increase in the defect-induced density ratios (*i.e. I*_D_/*I*_G_, *I*_TPA_/*I*_G_ (red circles) and *I*_A_/*I*_G_ (blue triangles)) with respect to the pristine SCS samples revealed a significant change in the number of defects due to the incorporation of the ions. With a prolonged ion implantation time of 14 h (Fig. 5b), a slight reduction in both *I*_TPA_/*I*_G_ and *I*_A_/*I*_G_ ratios for SCS-B^+^ and SCS-N^+^ samples indicated removal of the amorphous phases and/or adatoms, as well as possibly the re-ordering and/or removal of zig-zag chains at the shell edges due to the continuous bombardment with the ion beam. This resulted in improved sp^2^–sp^3^ ratio owing to the strong interactions of B and N with the carbon lattice through formation of stable sp^2^ bonds, hence the observed lower *I*_D_/*I*_G_ ratio, particularly for the SCS-N^+^ sample (∼1.22). The larger *I*_D_/*I*_G_ ratio (∼2.19) for the SCS-B^+^ sample could be attributed to the stabilization of B^+^ ions at the edges of the carbon shells, possibly in the form of B–O bonds,^50,51^ thereby increasing the amount of sp^3^-carbon species. In the case of the SCS-Ne^+^ sample, the prolonged implantation time led to an increased lattice distortion through generation of amorphous phases and polymeric zig-zag chains, highlighted by the increasing *I*_TPA_/*I*_G_ and *I*_A_/*I*_G_ ratios. However, the lower *I*_D_/*I*_G_ ratio (∼1.44) could be indicative of the restructuring of the carbon lattice of the carbon shells by the carbon adatoms and/or carbon radicals, subsequently resulting in an improved sp^2^–sp^3^ ratio. The complexity here merits further investigation.

### 3.3 Thermal stability

The thermal stability of the pristine and variously ion-implanted carbon spheres was investigated by the thermal gravimetric analysis (TGA) technique. Fig. S9 shows that the samples exhibited a similar, but not identical, TGA profile regardless of the implanted heteroatom. Furthermore, the complete decomposition of all samples was a confirmation that all the materials were predominantly carbon-based and contained little or no impurities.^52^ As expected, the implantation of the ions was observed to influence the thermal stability of the solid carbon spheres due to the formation of structural defective regions that facilitated the decomposition.^19^ For instance, the pristine SCSs showed a decomposition temperature of ∼584 °C, which was higher than that of the ion-implanted carbon structures. The lower onset decomposition temperatures for the 7 h implantation time (∼556–579 °C) in comparison to those for 14 h (∼563–581 °C) corroborated the Raman analysis data (Fig. 5a).

**Fig. 6:**
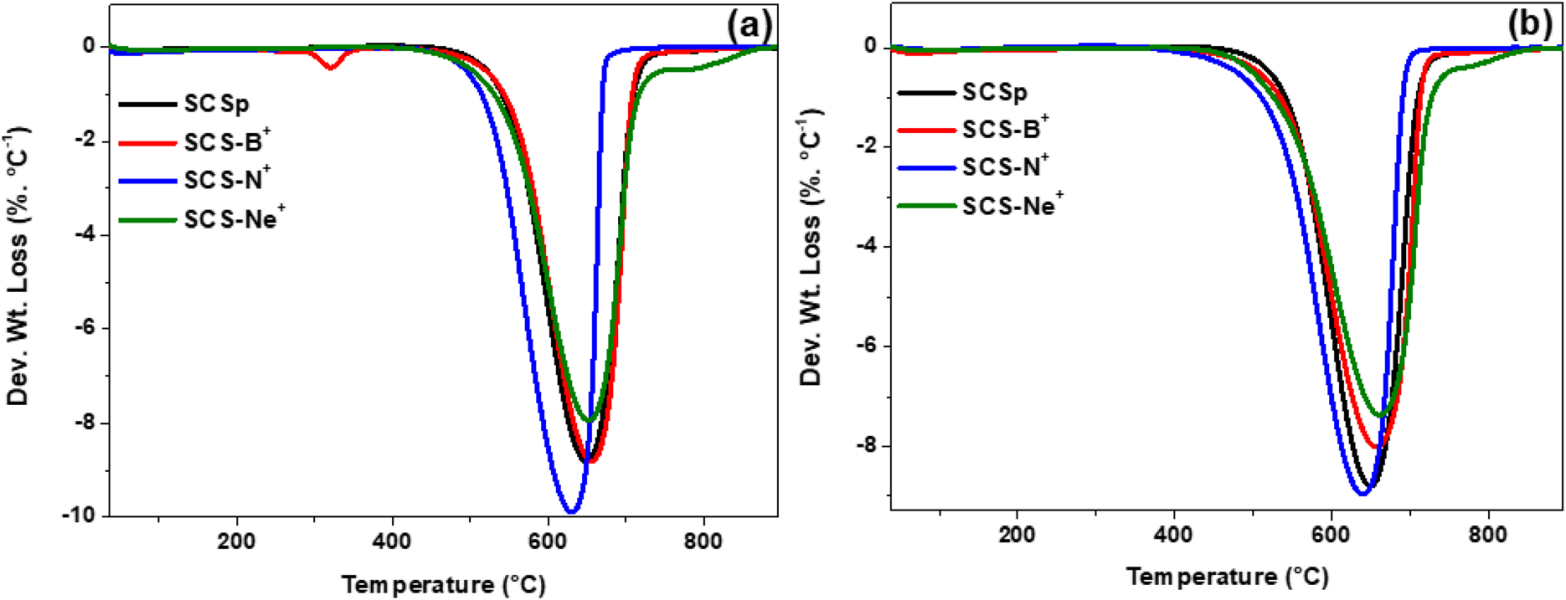
Derivative profiles of the SCSs samples after (a) 7 h and (b) 14 h implantation times.

The first order of the derivative thermogravimetry (DTG) weight loss profiles are shown in Fig. 6. For the SCS_p_ as well as the 7 h implanted SCS samples (Fig. 6a), a sharp and symmetrical decomposition peak was observed, thus indicating the homogenous decomposition of the highly defective structured nanospheres.^19^ The addition of defects associated with the implantation of the various ions had little effect as revealed in the narrow linewidth values of ∼93, ∼89, and ∼104 °C for the SCS-B^+^, SCS-N^+^, and SCS-Ne^+^ samples (Fig. S10b) respectively, compared with SCS_p_ which is 96 °C (Fig. S10b, blue squares). On the other hand, the prolonged implantation time of 14 h gave samples that exhibited slightly broader decomposition peaks with the linewidth range of ∼99–115 °C (Fig. S10b, green diamonds). The creation of fewer defective regions as suggested by the Raman analysis data (Fig. 5b) may have resulted in a restoration of the outer carbon region, thereby leading to more thermally stable carbonaceous materials as shown by the decomposition values of ∼656, ∼639, and ∼660 °C (Fig. S10a) for the SCS-B^+^, SCS-N^+^, and SCS-Ne^+^ samples, respectively.

### 3.4 Textural properties

**Fig. 7:**
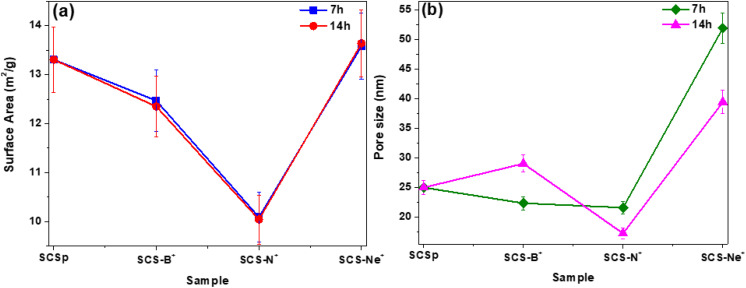
(a) BET surface area and (b) pore size of the pristine and ion-implanted carbon spheres. The connecting lines serve only as a guide for the eye.

Changes in the specific surface areas (SSA) of the carbons as a function of the type of the ion and the fluence time were determined using the multi-point Brunauer–Emmett–Teller (BET)^53,54^ method, whilst the pore size distributions were determined using the Barrett–Joyner–Halenda (BJH)^55^ method. The N_2_ adsorption/desorption isotherm curves of all samples (Fig. S11) demonstrated a typical type (II) isotherm, corresponding to macroporous or relatively non-porous materials exhibiting a monolayer adsorption of N_2_ onto the surface of the solid carbon spheres. Determination of the surface areas from the multi-point BET method (Fig. 7a) showed that, regardless of the implantation time, the samples exhibited very low surface areas (∼10–13.5 m^2^ g^−1^). However, the lower surface area for the SCS-B^+^ (∼12.5 m^2^ g^−1^) and SCS-N^+^ (∼10.5 m^2^ g^−1^) samples in comparison to the SCS_p_ (∼13.3 m^2^ g^−1^), may be attributed to the good affinity of carbon for both boron and nitrogen atoms. This was also suggested by the slight to no reduction in the pore volume and pore sizes (Fig. 7b). On the other hand, the slightly increased surface area for the SCS-Ne^+^ samples can be attributed to the radiation damage caused by these heavier ions. This also led to almost a twofold enlargement of the pores (Fig. 7b).

### 3.5 Magnetic moment properties

**Fig. 8:**
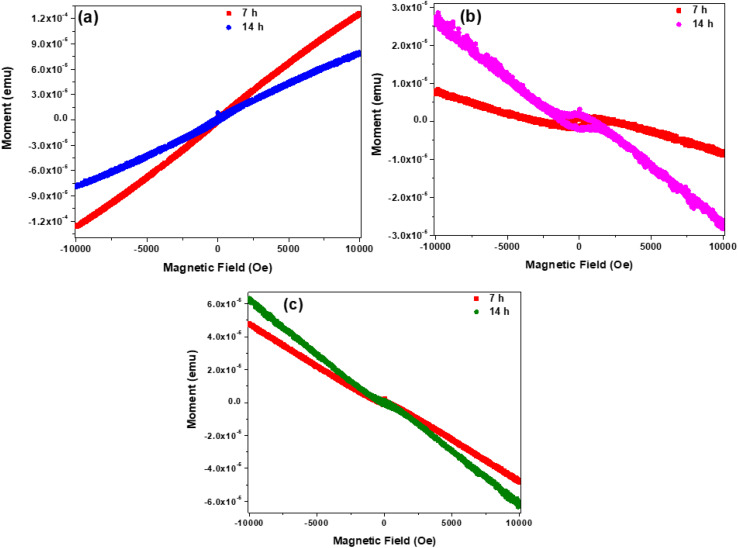
Isotherm curves of the SCS-Ne^+^ samples after different irradiation times at (a) 2 K, (b) 10 K and (c) 40 K.

The influence of implantation of heteroatom ions on the magnetic properties of the solid carbon nanospheres was investigated by determining the isotherms under increasing magnetic field and at different temperatures. The isotherms of the baseline SCS-Ne^+^ samples after 7 h and 14 h implantation times are shown in Fig. 8, whilst those of SCS-N^+^ and SCS-B^+^ samples are presented in Fig. S12 and S13, respectively. For both the 7 h and 14 h implanted SCS-Ne^+^ samples, a magnetic structural transition from the usual diamagnetism to super-paramagnetism at low temperature (<10 K) is observed (Fig. 8). This suggests that the ∼200 nm particles are small enough to contain single aligned domains. However, the paramagnetic centres are thermally deactivated by temperature (Fig. 8b and c). Both the dia- and paramagnetic susceptibilities depend only weakly on the fluence of Ne^+^ except around the transition temperature (Fig. 8b); contrast with Fig. S13.

Susceptibility values for all three ions are listed in Table 1, and these diverge strongly when electrically active ions are implanted. Values (absolute) are about 1 × 10^−8^ for Ne^+^ and N^+^ for both the diamagnetic and superparamagnetic magnetizing slopes but the latter, in the case of boron, rises to 2.6 × 10^−8^ for the lower fluence and to 3.8 × 10^−7^ for the 14 h implant – an increase of nearly 40-fold. Clearly the B dopant is contributing to unpaired spins or other electrically active centres, and the effect is progressive.

**Table 1:** Susceptibility values taken from Fig. 8, S12 and S13, plotted in emu. Rough estimates of the Néel temperatures read from Fig. 9, S14 and S15 are also included

Implant	Susceptibility (2 K)	Susceptibility (40 K)	Néel temperature
Boron – 7 h	2.6 × 10^−8^	−7 × 10^−9^	∼8 K
Boron – 14 h	3.8 × 10^−7^	−8 × 10^−9^	∼10 K
Nitrogen – 7 h		−1.1 × 10^−8^	∼14 K
Nitrogen – 14 h	1.2 × 10^−8^	−1.1 × 10^−8^	∼5 K
Neon – 7 h	1.3 × 10^−8^	−5 × 10^−9^	∼6 K
Neon – 14 h	8 × 10^−9^	−6 × 10^−9^	∼4 K

**Fig. 9:**
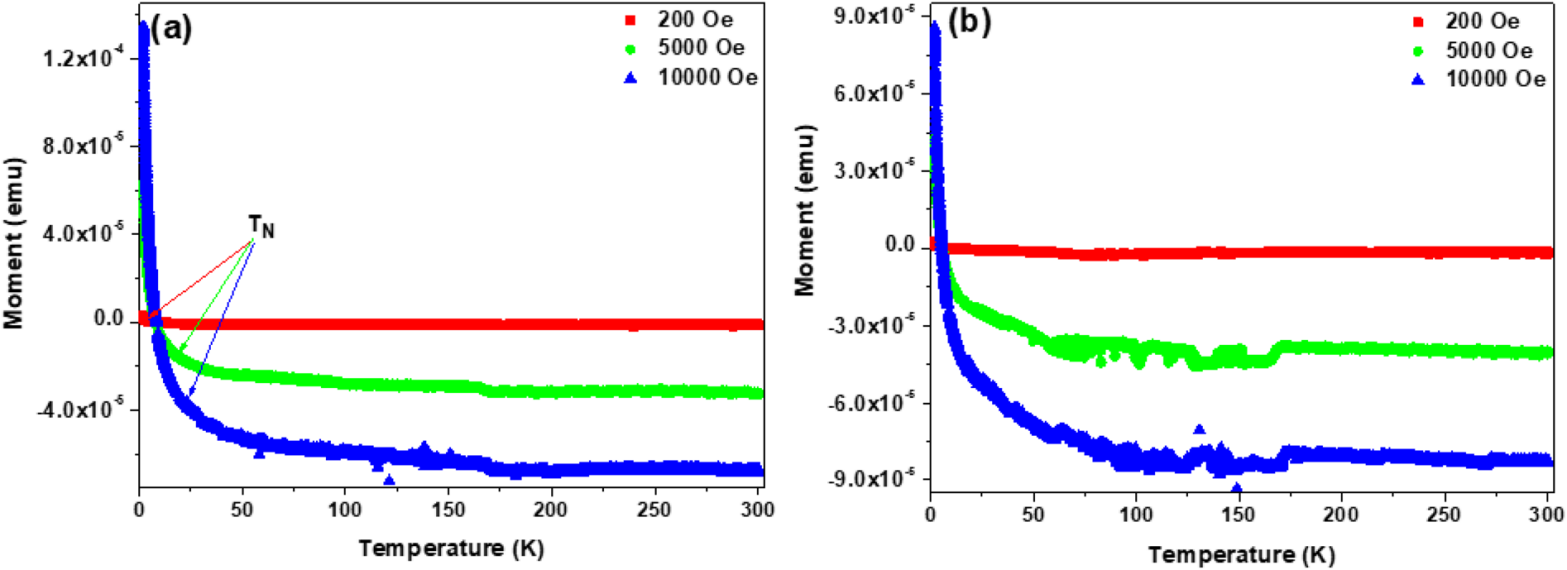
Isofield (*M*–*T*) measurements for the SCS-Ne^+^ samples after (a) 7 h and (b) 14 h implantation times.

The Néel transition temperatures were determined by plotting the isofield (*M*–*T*) curves (Fig. 9 for Ne^+^, Fig. S14 for N^+^ and Fig. S15 for B^+^). This is further illustrated by the field cooled and zero field cooled curves of the samples upon taking isofield measurements in the scan from 300 to 2 K. Typically, the Néel temperature occurs when the thermal energy destroys the exchange-mediated antiferromagnetic ordering, transforming it into a diamagnetic material.^56,57^ For the 7 h SCS-Ne^+^ sample, it is clear that the Néel temperature is field dependent and occurs at *T* < 40 K (Fig. 9a). On the other hand, *M*–*T* curves of the 14 h SCS-Ne^+^ samples (Fig. 9b) illustrated that application of a stronger field enhanced the diamagnetic response as well as the curvature of the *M*–*T* curves at low temperature. Similar results occur for N^+^ and B^+^ (Fig. S14 and S15 respectively). The magnetization was seen to assume a positive value at *T* < 10 K thereby confirming the presence of super paramagnetic centres and enabling the Néel temperatures to be estimated, as listed in Table 1.

### 3.6 Electrical conductivity

Attempts to measure the conductivity or resistivity, especially of the B^+^ doped samples, by variants of the van der Pauw technique, have proven to be very challenging on the powder samples. A crude estimate for the B^+^ samples after dispersing 20 mg of SCS's in 500 ml of distilled water through ultrasonication, then filtering the suspension through a polytetrafluoroethylene (PTFE) membrane filter paper using vacuum filtration and cutting the film into 0.5 cm × 0.5 cm samples, was made. Sheet resistances were determined by pressing four electrodes on to the nanosphere film (Table 2). The sheet resistance decreased strongly with increasing implant fluence, showing a strong temperature effect, suggesting that boron acceptor atoms were incorporated within the carbon structures. More work remains to be done if the problems of using powder samples can be solved.

**Table 2:** Resistance response of B^+^ samples as function of temperature, in ohms. Error bars could not be determined

Temperature (°C)	Ω		
	Pristine	7 h	14 h
250	17 220	8674	6013
200	25 580	13 083	9213
150	41 290	19 794	18 108
100	89 610	46 775	36 537
50	583 800	438 599	390 548

## 4. Conclusions

The influence of implantation of different ions into solid carbon nanospheres of *ca.* 200 nm diameter on their physicochemical and electronic properties was investigated. The ions studied were B^+^, N^+^ and Ne^+^ and the dosage of these ions into the carbon was controlled by varying the implantation time (7 h and 14 h), to give uniform ion loaded levels of approximately 300 ppm and 600 ppm respectively. The morphological studies showed that the accreted nature of the solid carbon spheres was retained post implantation after different ion beam exposure times. Additionally, Raman data showed that all the synthesized carbon spheres exhibited a low degree of graphitization as characterized by the large values for the defect density ratios of *I*_D_/*I*_G_, *I*_TPA_/*I*_G_, and *I*_A_/*I*_G_. Furthermore, both the textural and thermal stability studies showed modest changes in the influence of the different ions on these properties of the carbon spheres. Magnetic properties of the solid carbon spheres were found to be dependent on the type of ion implanted, with a diamagnetic to superparamagnetic transition seen below *ca.* 10 K, and a much higher susceptibility in the case of B^+^, that increased with fluence. An attempt to confirm boron doping using electrical conductivity showed the expected behaviour, with the resistance dropping sharply with increasing B^+^ fluence. Generally, the study indicated the importance of using different heteroatom ions on modifying the properties of solid carbon spheres. This suggests an avenue for the design of making modern carbon-based electromechanical devices using doping ion implantation processes. It is important that the doping process used does not destroy the carbon structure but only modifies the carbon properties.

## Author contributions

TD: project conception, data analysis (ion implantation data collection *etc.*, magnetic susceptibilities), writing of manuscript. NJC: project conception, writing of manuscript. RE: Raman data collection and analysis. DM: conductivity data collection and analysis. KR: carbon synthesis, data collection and analysis. JBM: manuscript preparation, data collection.

## Conflicts of interest

There are no conflicts to declare.

## Supplementary Material

NA-007-D5NA00616C-s001

NA-007-D5NA00616C-s002

NA-007-D5NA00616C-s003

NA-007-D5NA00616C-s004

NA-007-D5NA00616C-s005

NA-007-D5NA00616C-s006

NA-007-D5NA00616C-s007

NA-007-D5NA00616C-s008

NA-007-D5NA00616C-s009

NA-007-D5NA00616C-s010

NA-007-D5NA00616C-s011

NA-007-D5NA00616C-s012

NA-007-D5NA00616C-s013

NA-007-D5NA00616C-s014

NA-007-D5NA00616C-s015

NA-007-D5NA00616C-s016

NA-007-D5NA00616C-s017

## Data Availability

The data is available on request from the authors. Supplementary information is available. See DOI: https://doi.org/10.1039/d5na00616c.
