# Supplementary figures and images for: Modulating properties of solid carbon nanospheres via ion implantation with hetero-ions
Source: Nanoscale Adv. 2025 Aug 28;7(20):6451–61. doi: 10.1039/d5na00616c (PMC12421429; doi:10.1039/d5na00616c)

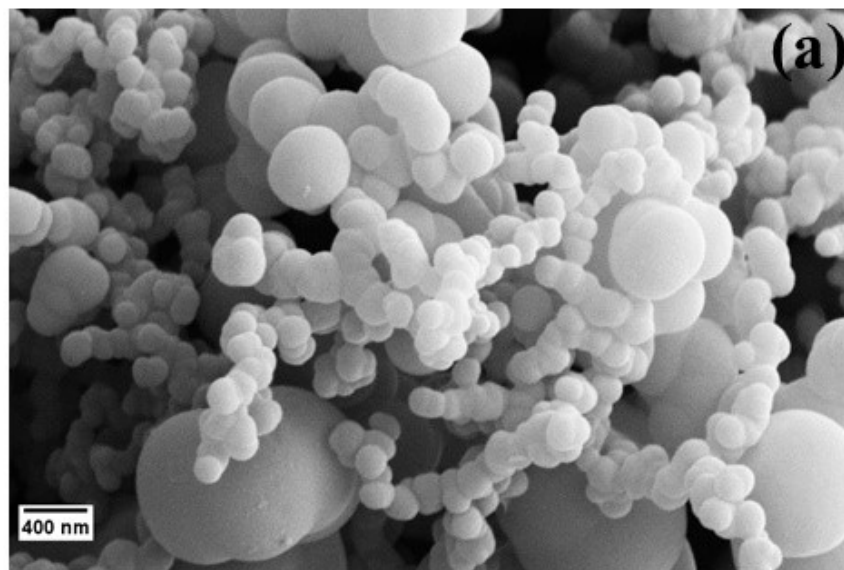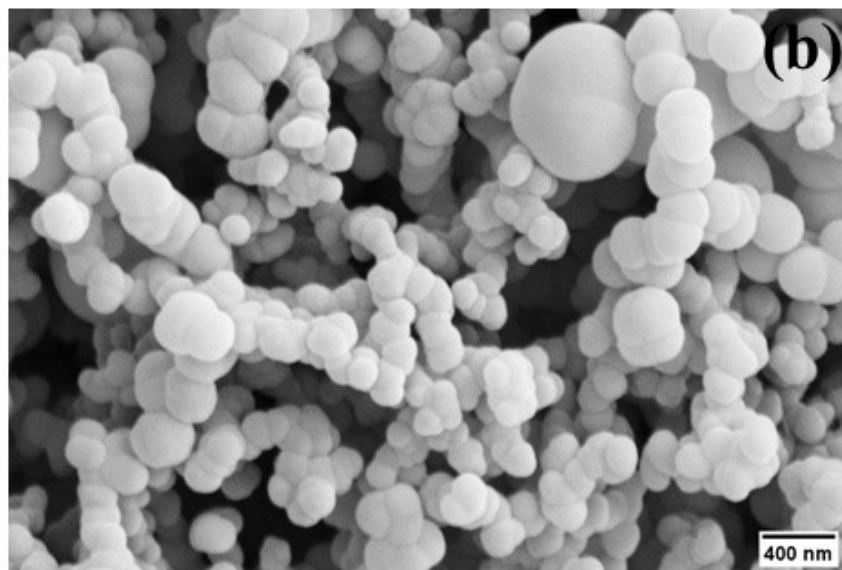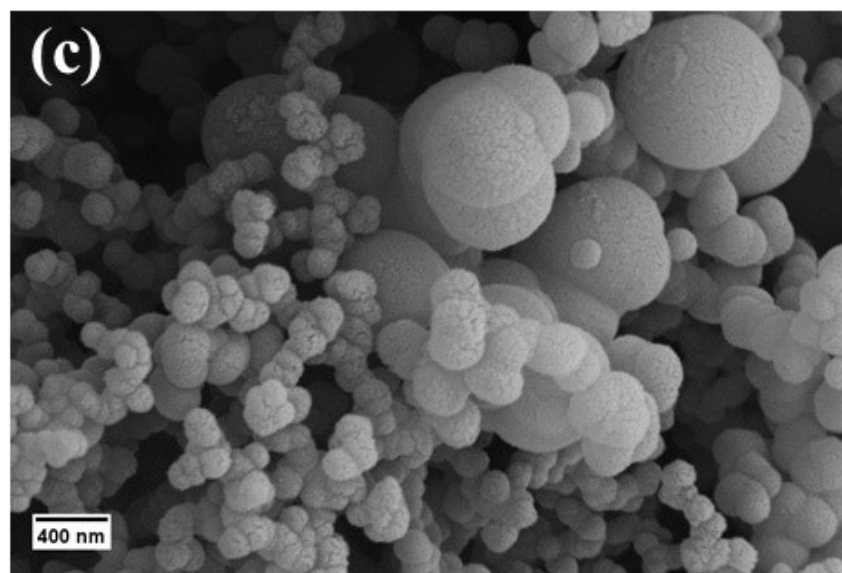

Supplement: NA-007-D5NA00616C-s002 [file NA-007-D5NA00616C-s002.pdf]

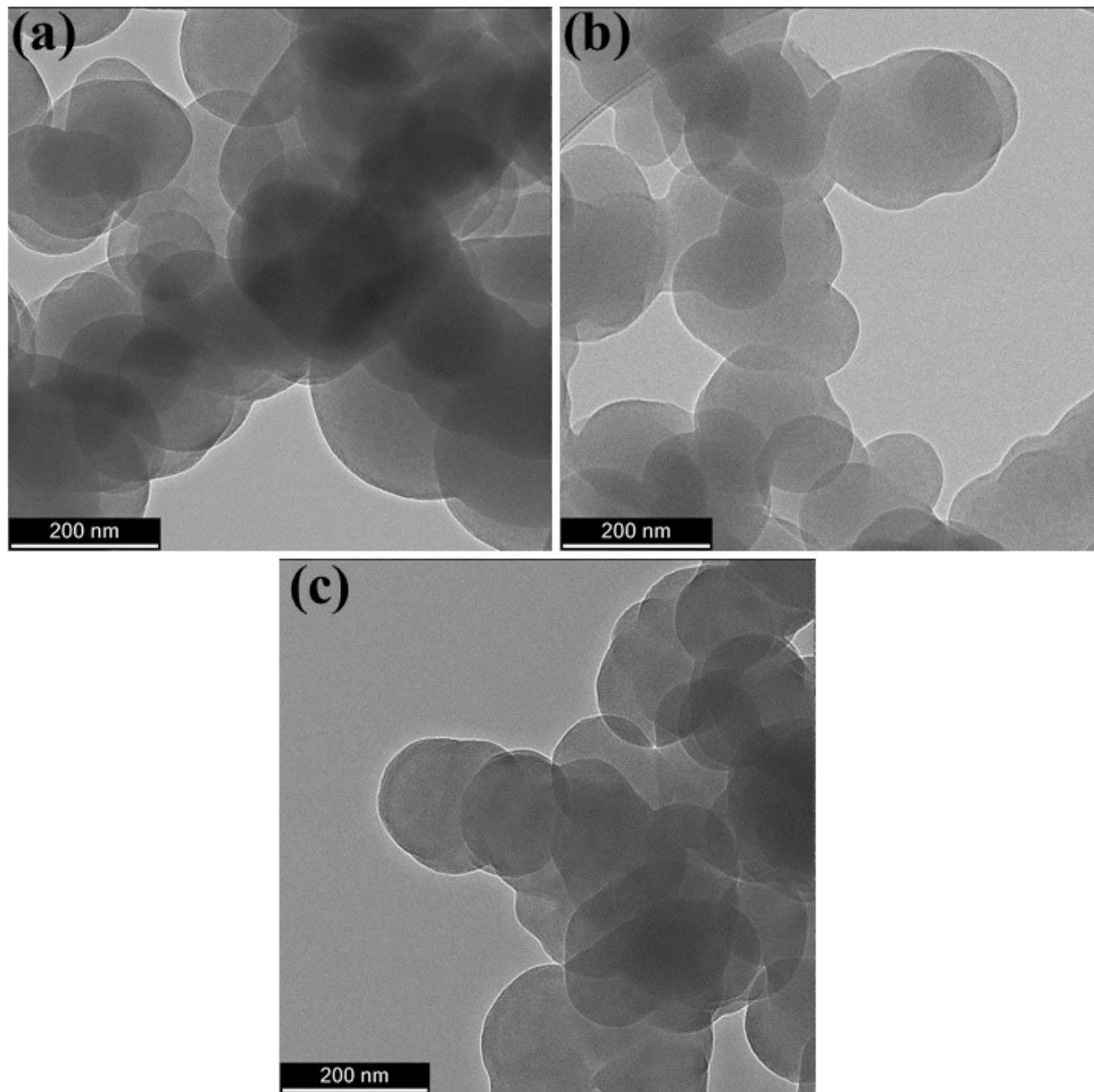

Supplement: NA-007-D5NA00616C-s003 [file NA-007-D5NA00616C-s003.pdf]

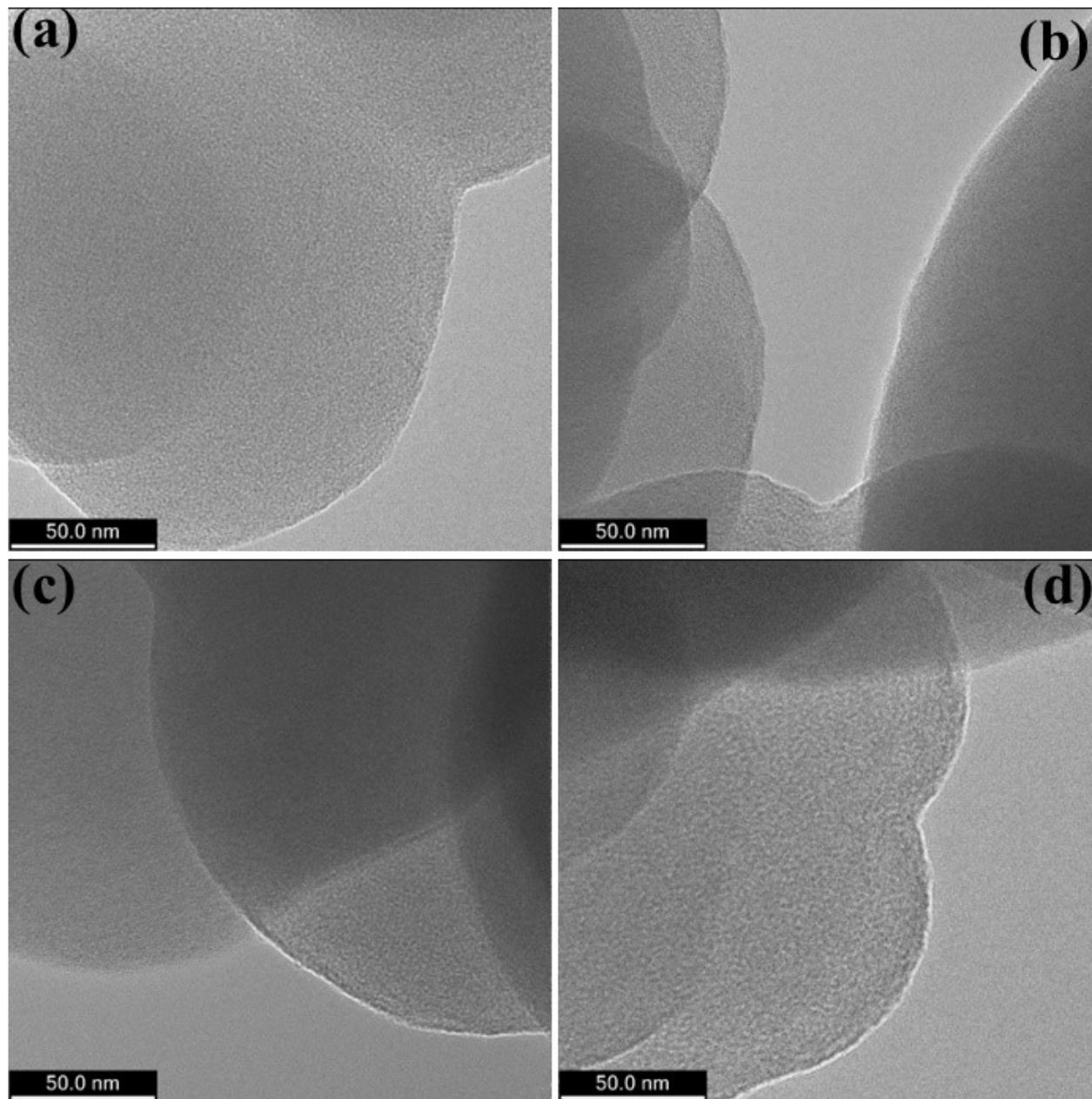

Supplement: NA-007-D5NA00616C-s004 [file NA-007-D5NA00616C-s004.pdf]

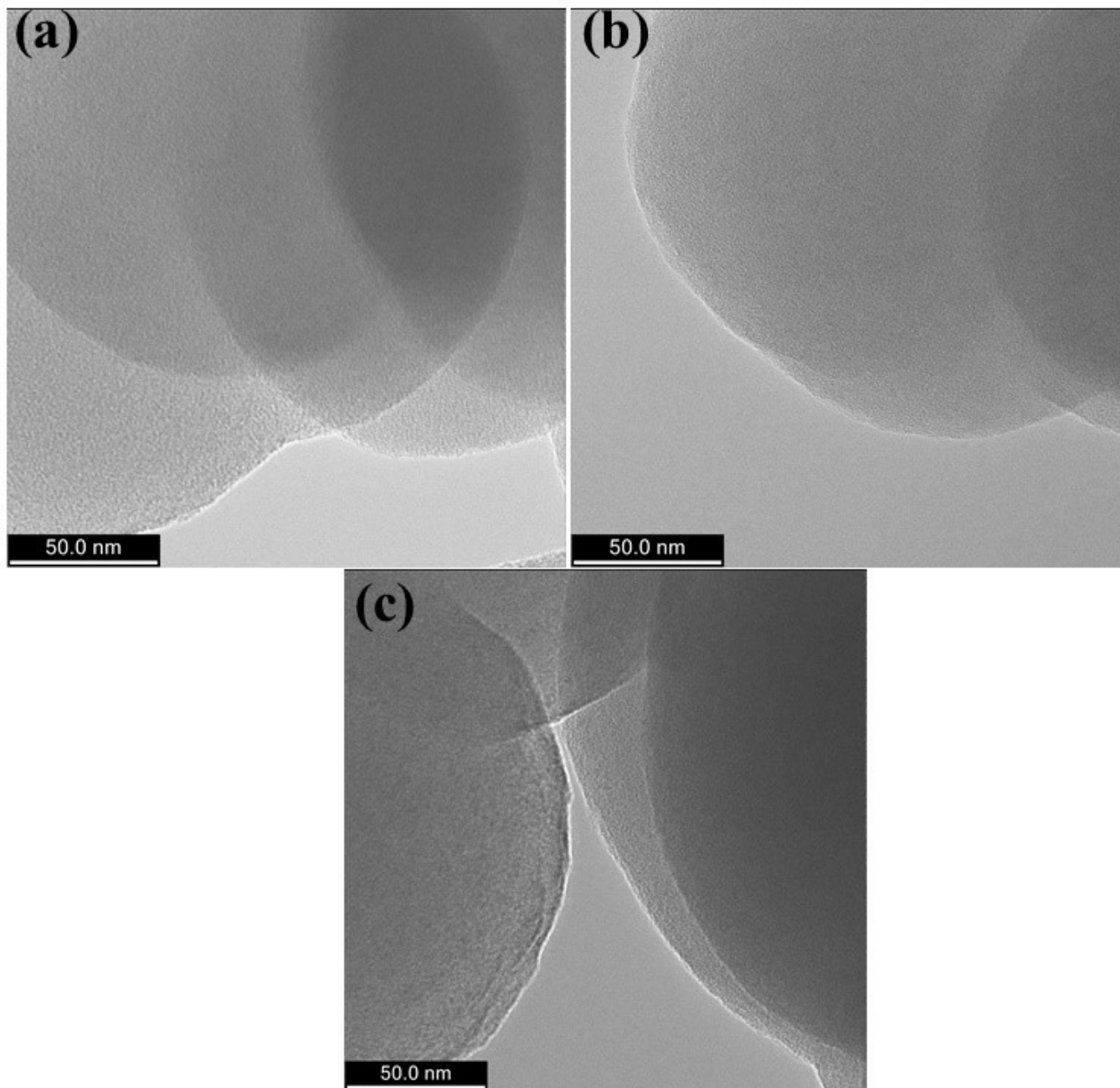

Supplement: NA-007-D5NA00616C-s005 [file NA-007-D5NA00616C-s005.pdf]

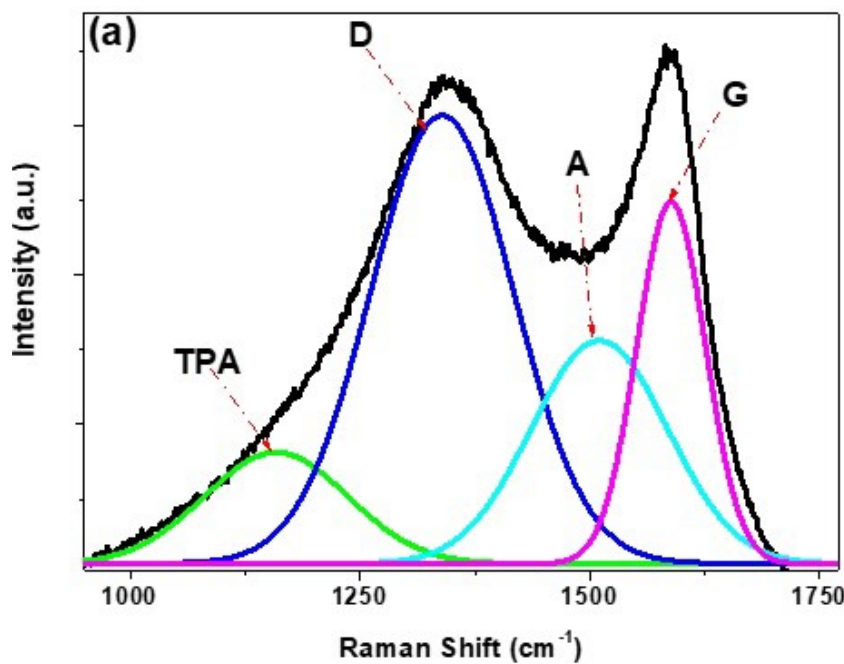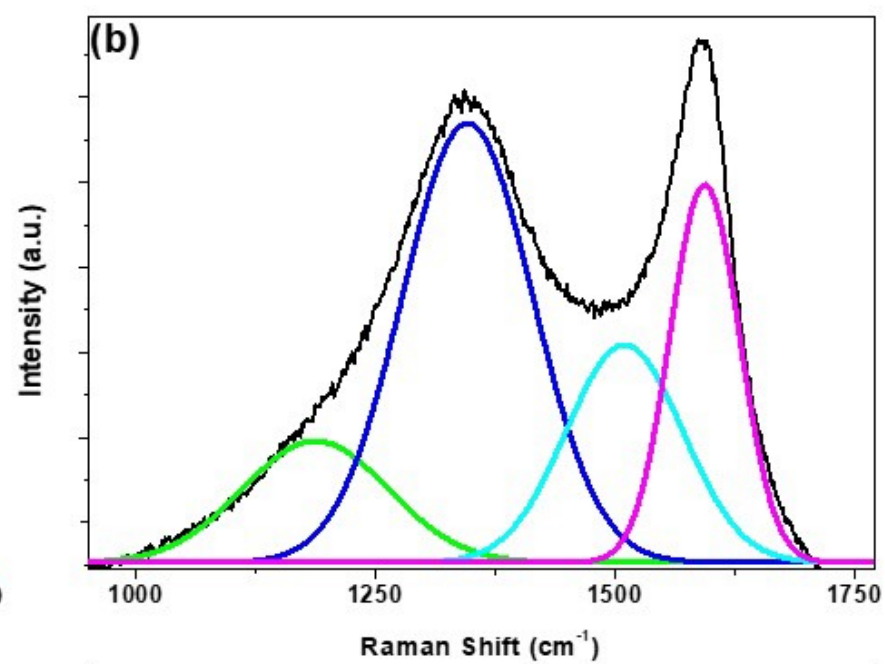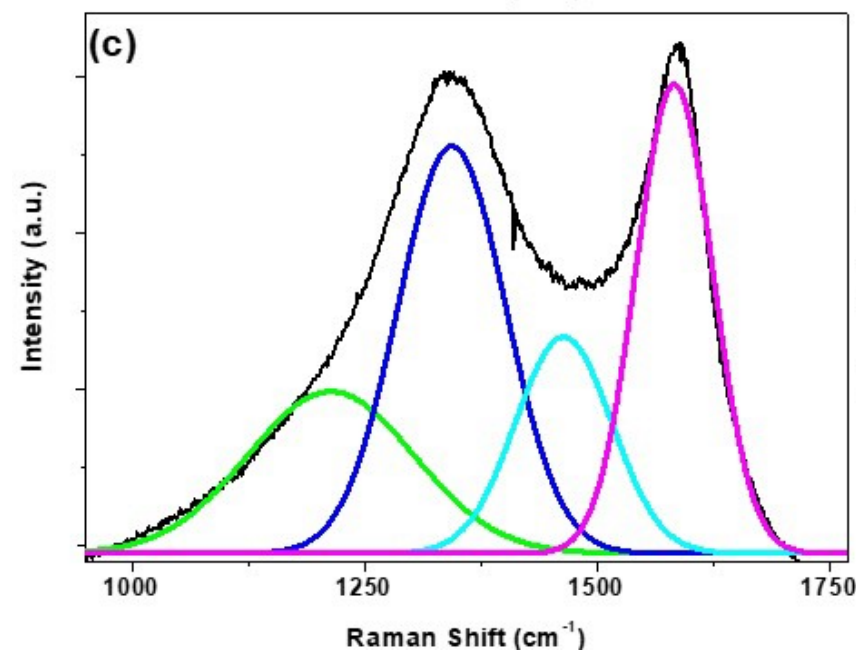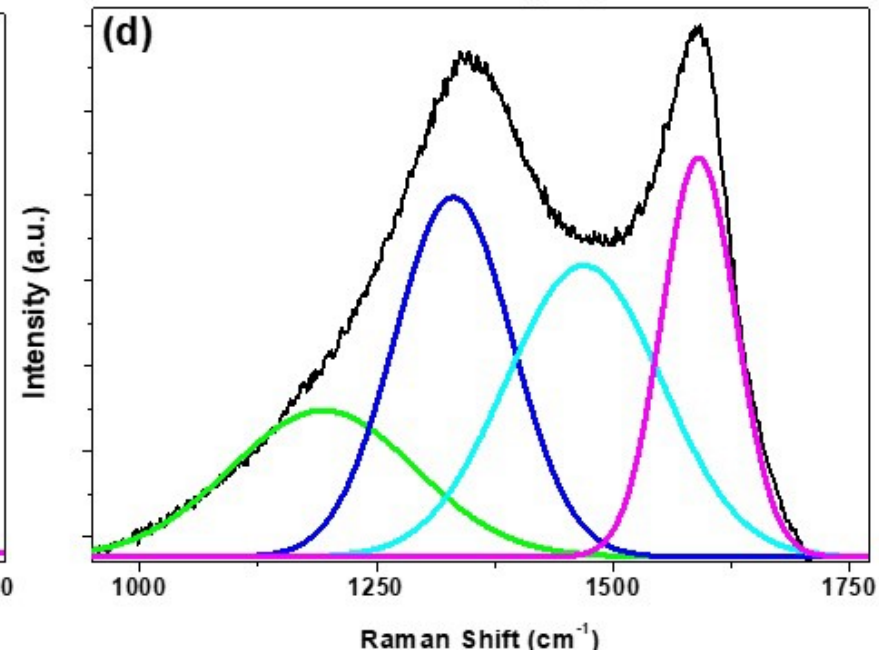

Supplement: NA-007-D5NA00616C-s006 [file NA-007-D5NA00616C-s006.pdf]

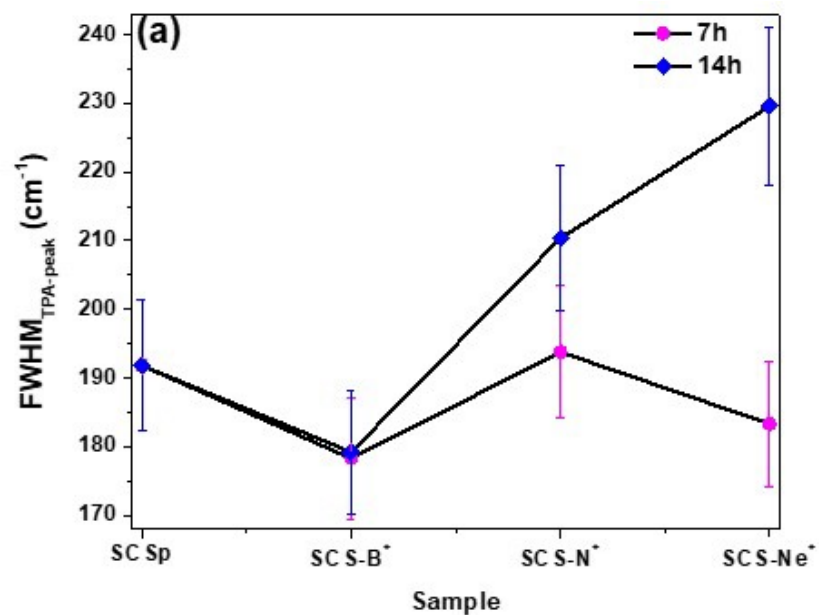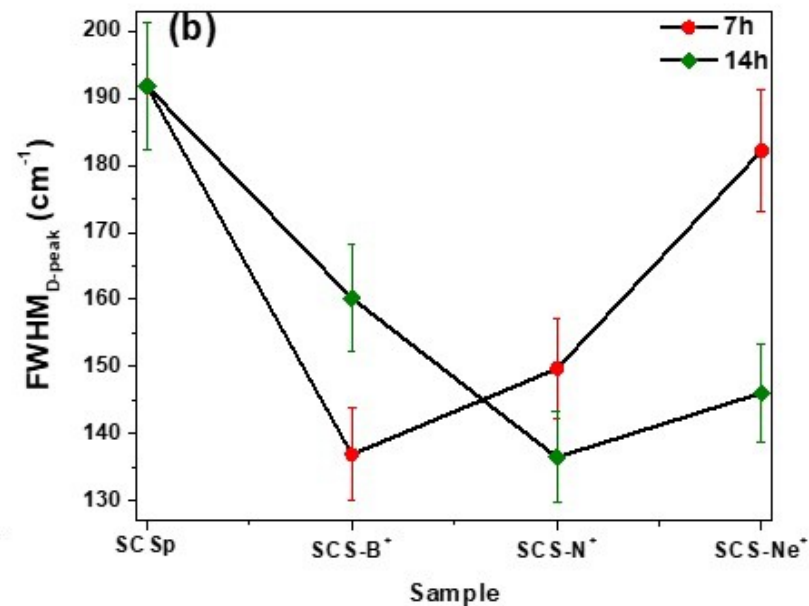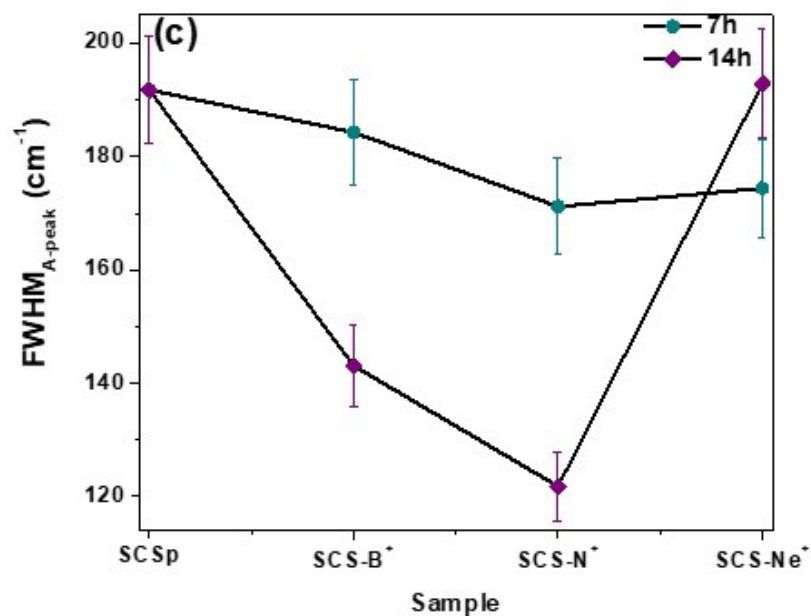

Supplement: NA-007-D5NA00616C-s007 [file NA-007-D5NA00616C-s007.pdf]

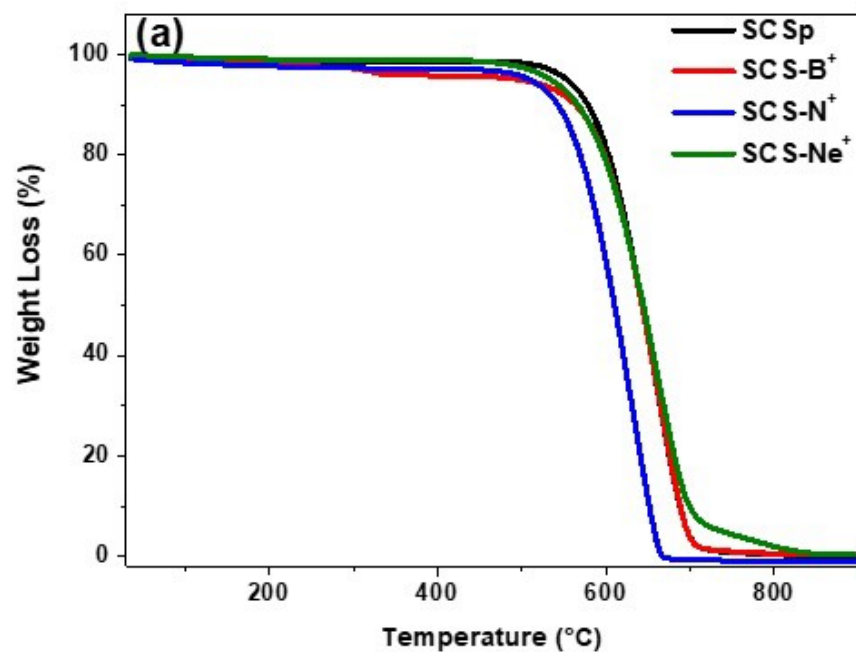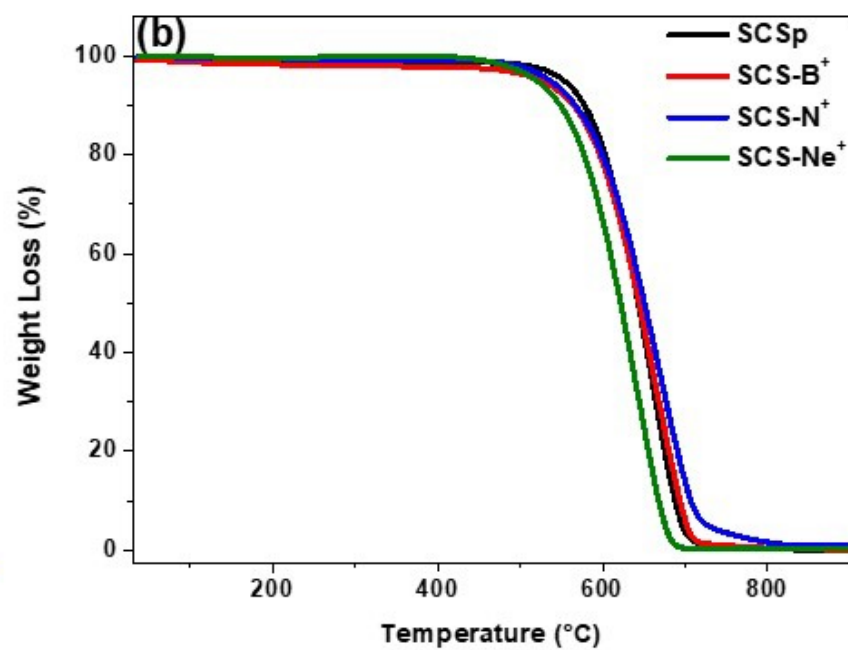

Supplement: NA-007-D5NA00616C-s008 [file NA-007-D5NA00616C-s008.pdf]

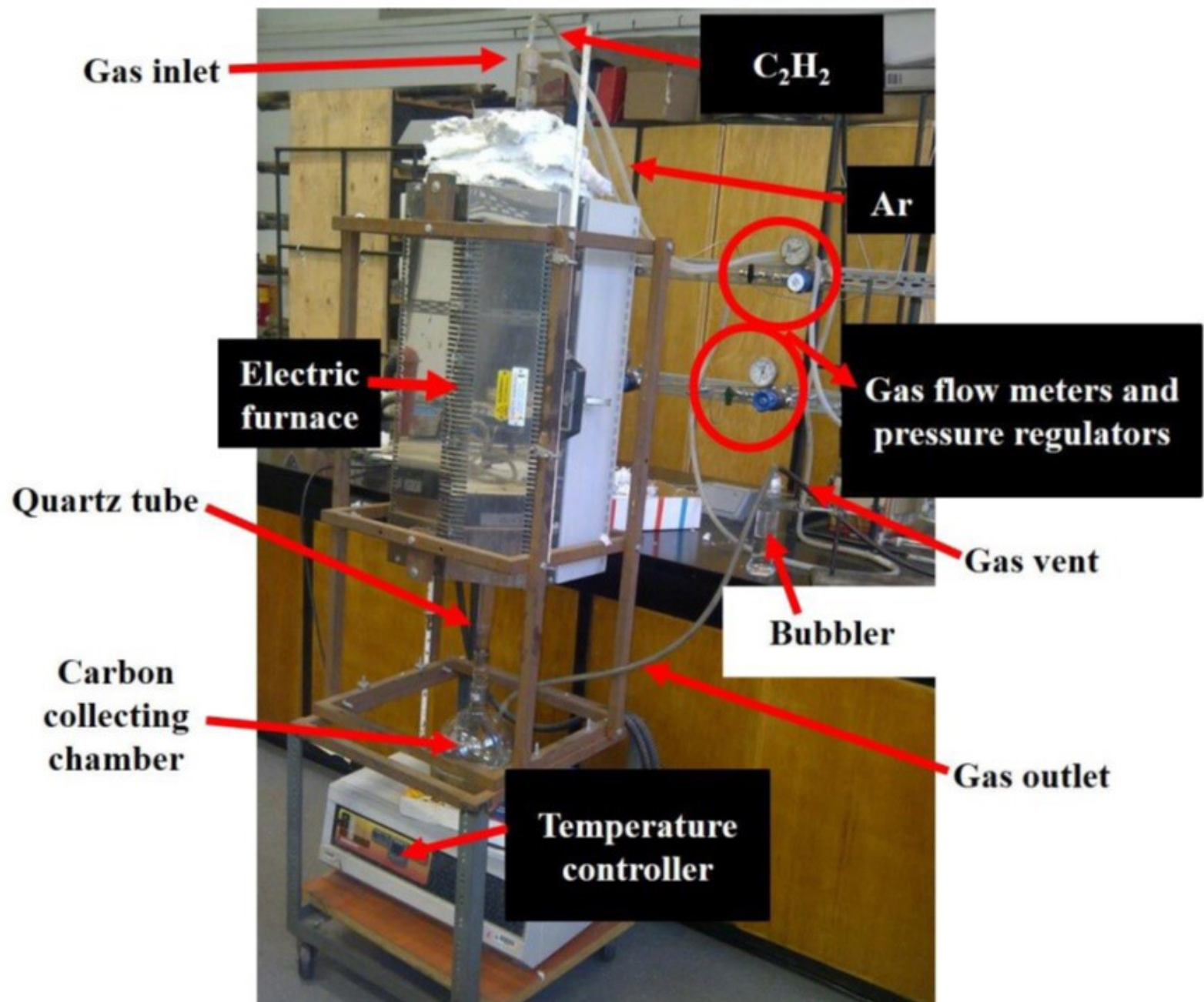

Supplement: NA-007-D5NA00616C-s010 [file NA-007-D5NA00616C-s010.pdf]

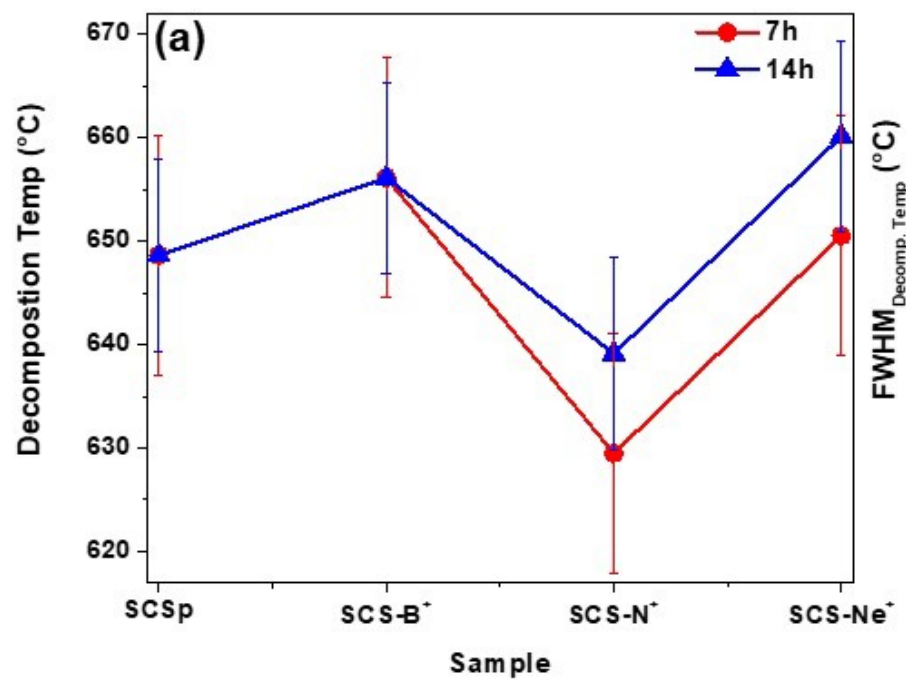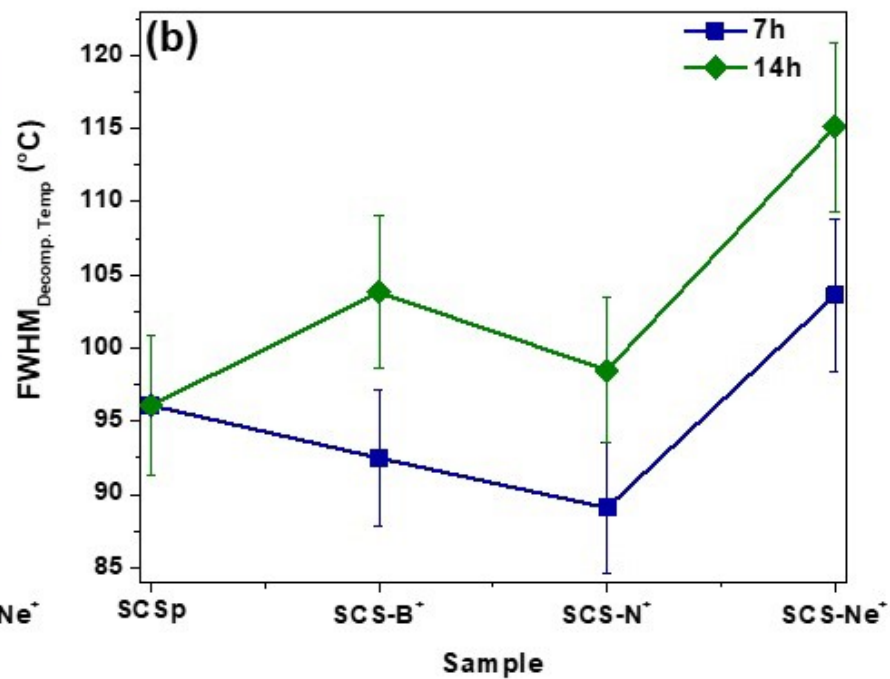

Supplement: NA-007-D5NA00616C-s011 [file NA-007-D5NA00616C-s011.pdf]

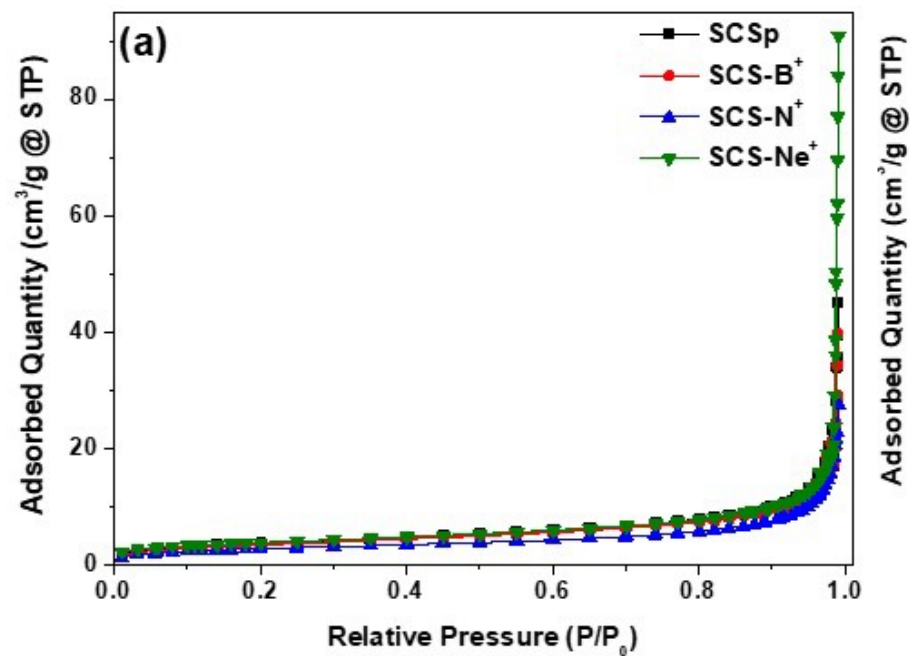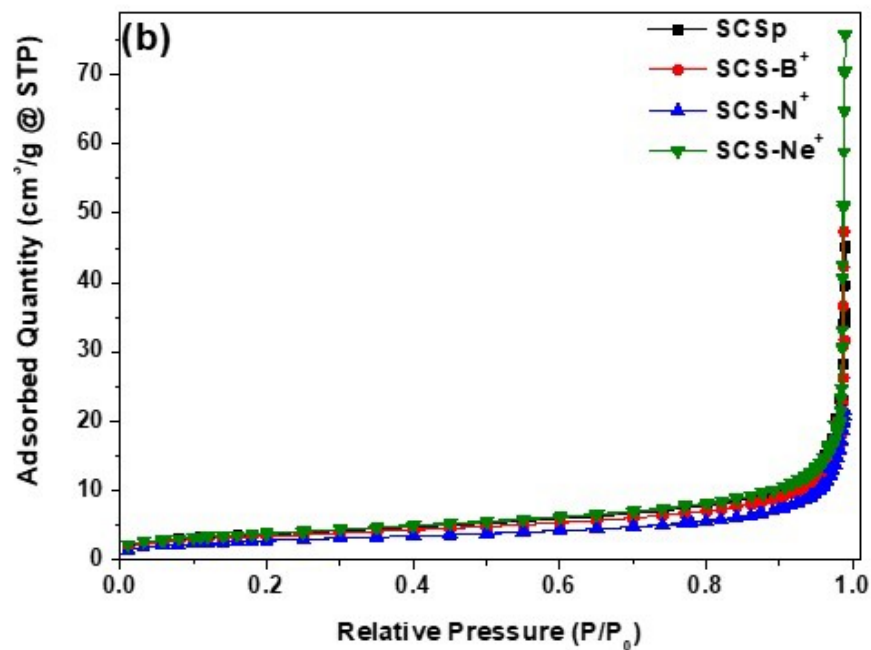

Supplement: NA-007-D5NA00616C-s012 [file NA-007-D5NA00616C-s012.pdf]

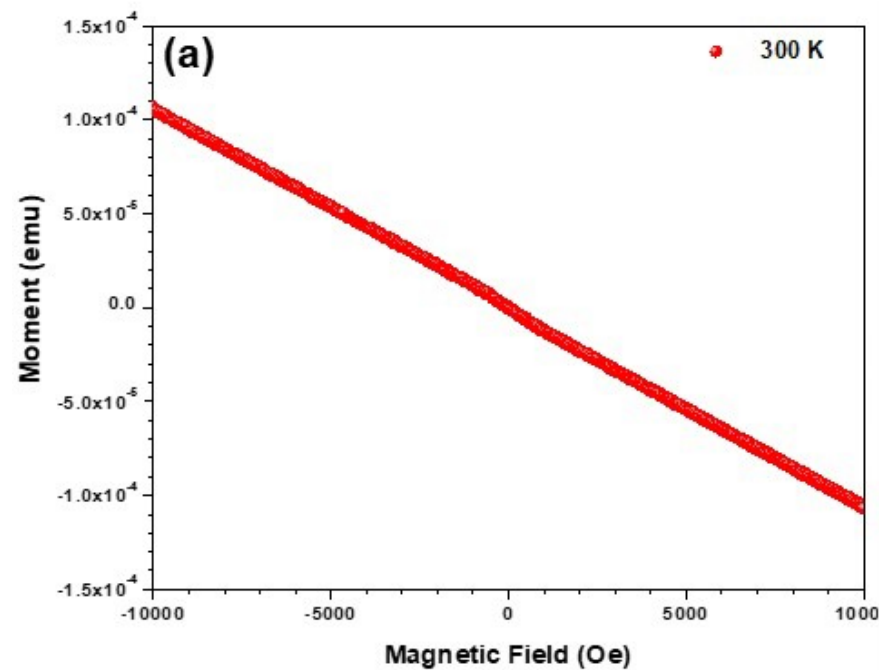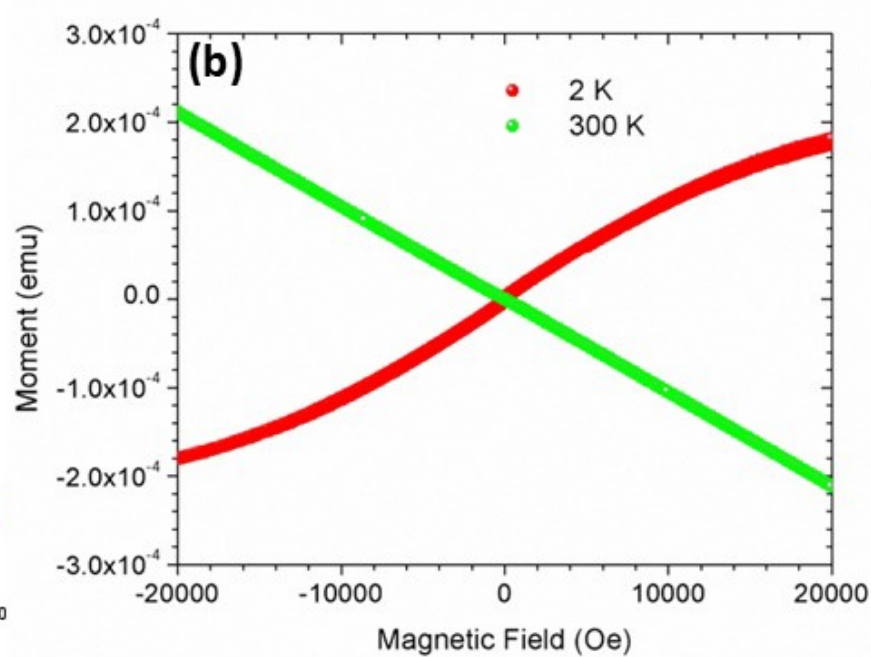

Supplement: NA-007-D5NA00616C-s013 [file NA-007-D5NA00616C-s013.pdf]

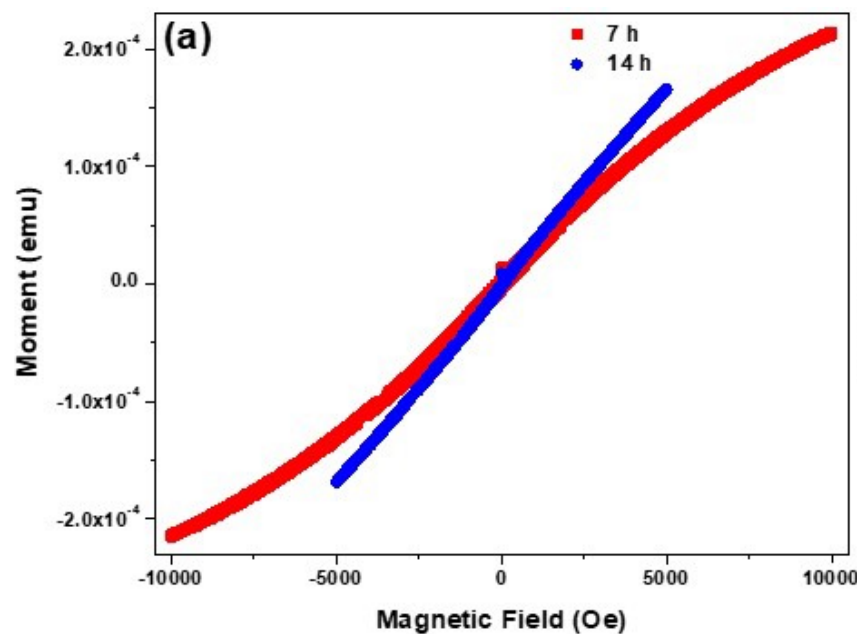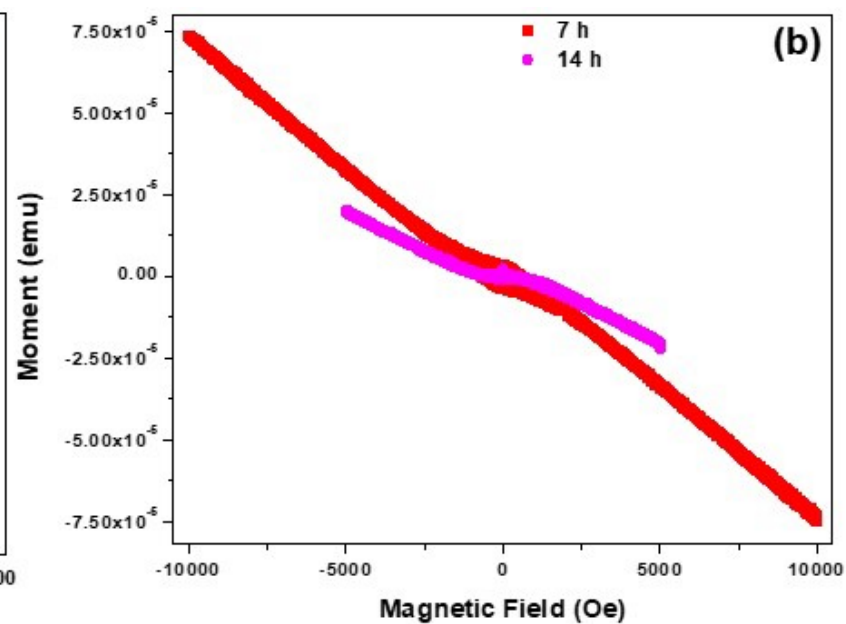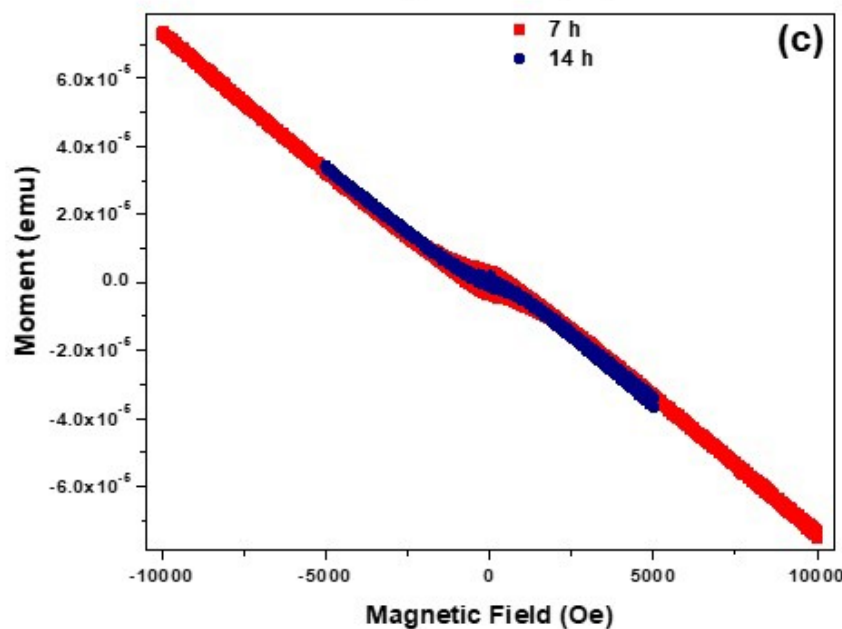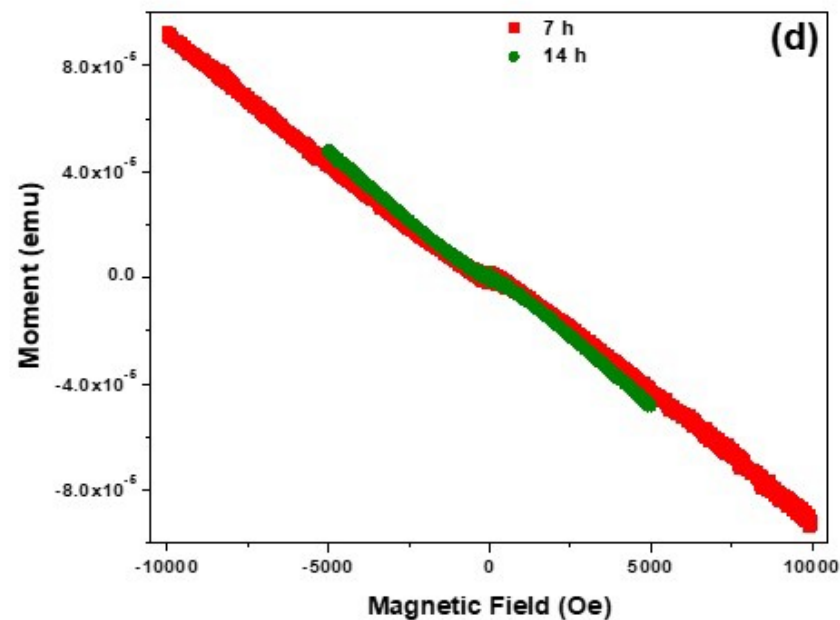

Supplement: NA-007-D5NA00616C-s014 [file NA-007-D5NA00616C-s014.pdf]

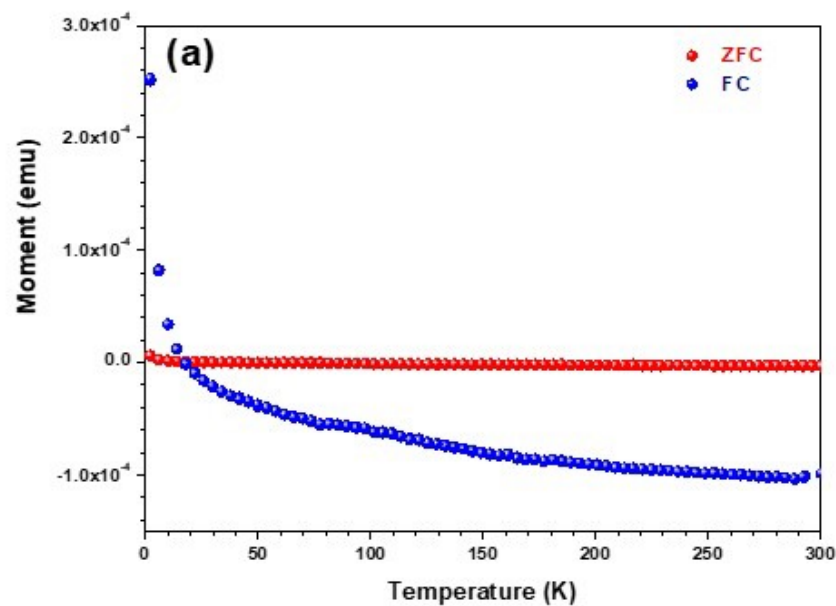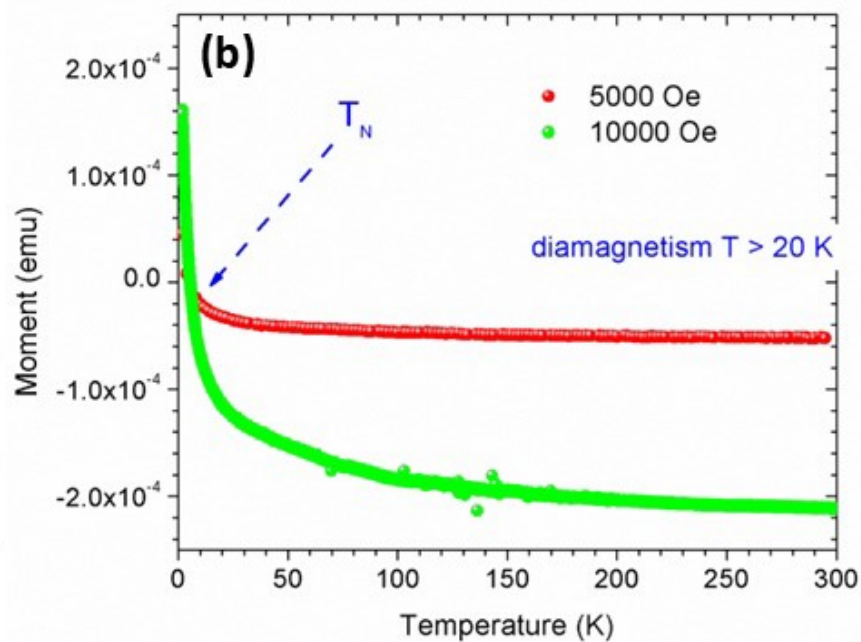

Supplement: NA-007-D5NA00616C-s015 [file NA-007-D5NA00616C-s015.pdf]

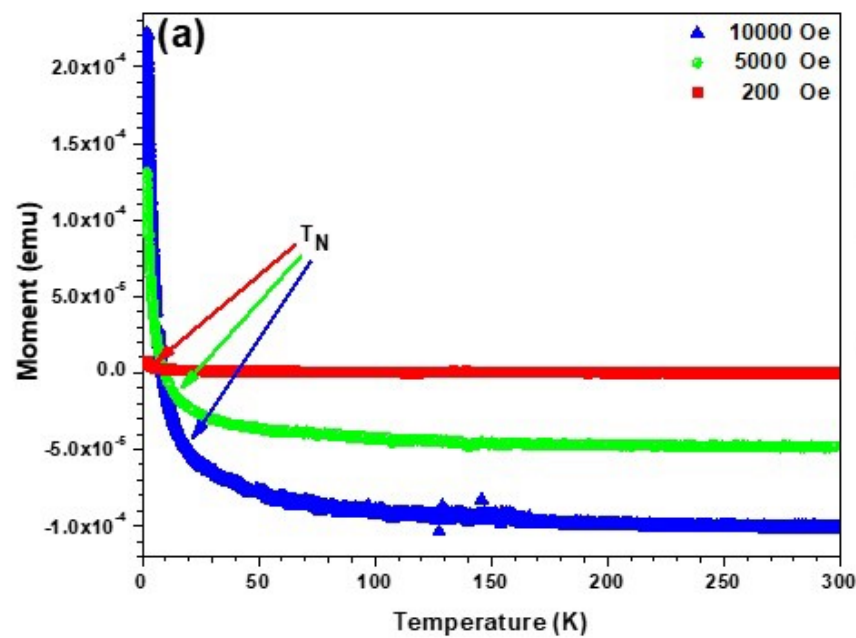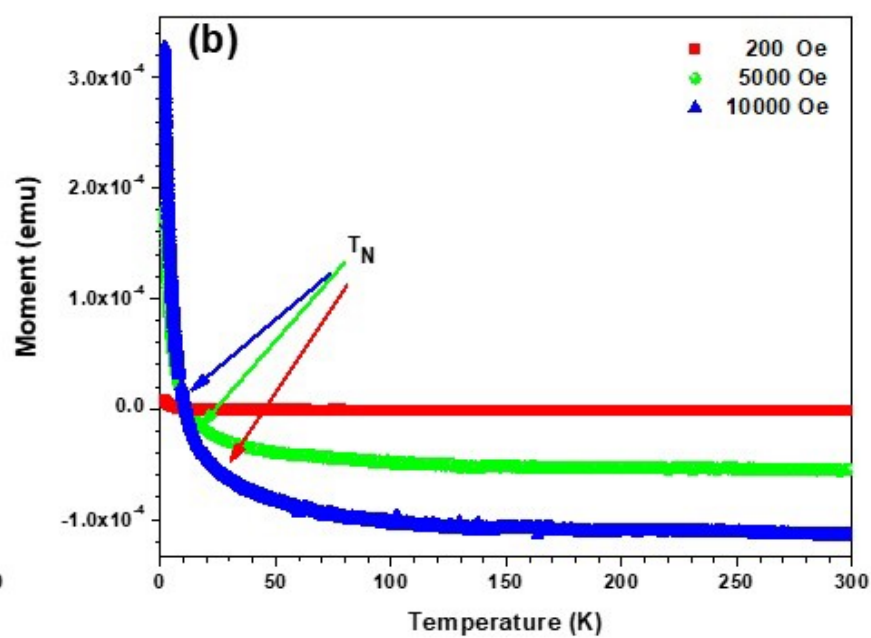

Supplement: NA-007-D5NA00616C-s016 [file NA-007-D5NA00616C-s016.pdf]

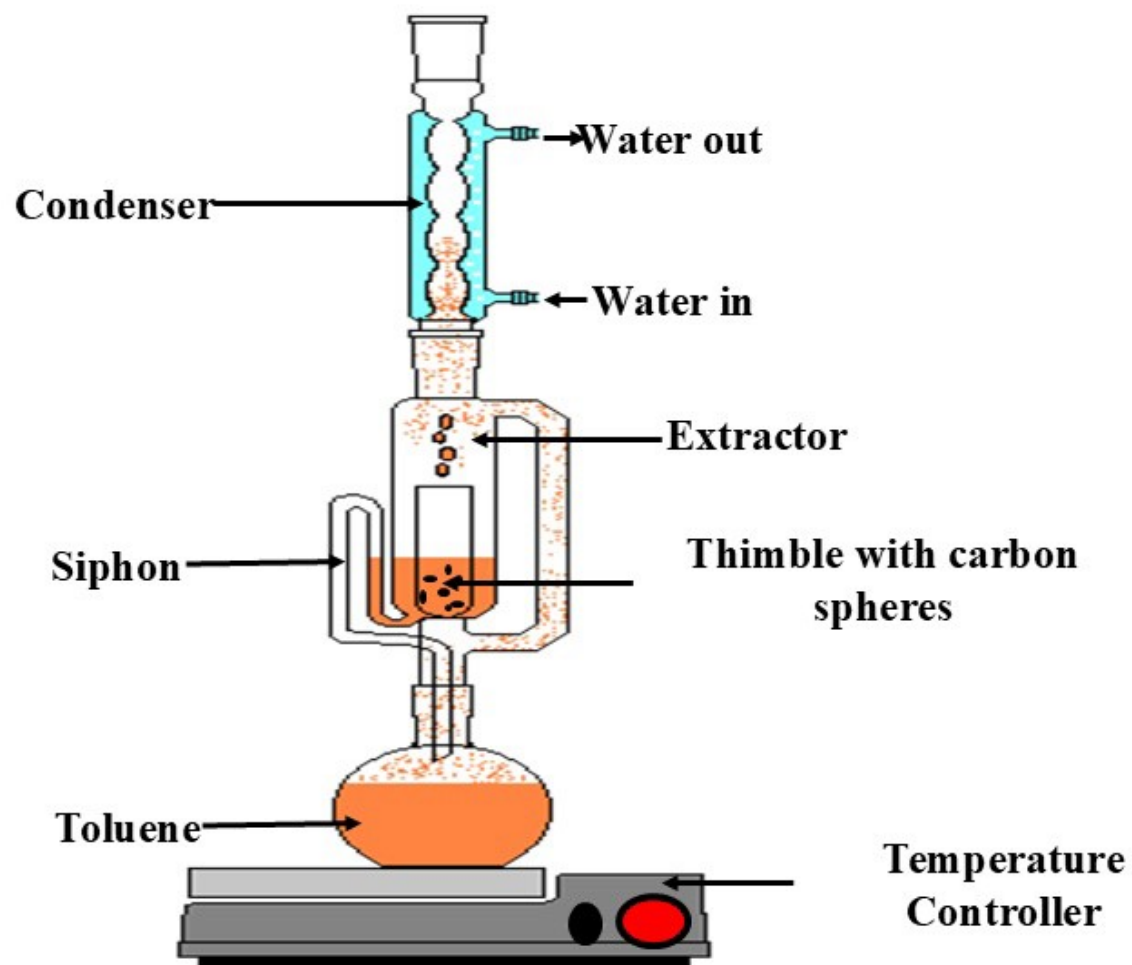

Supplement: NA-007-D5NA00616C-s017 [file NA-007-D5NA00616C-s017.pdf]
