# Supplementary material for: Modulating properties of solid carbon nanospheres via ion implantation with hetero-ions
Source: Nanoscale Adv. 2025 Aug 28;7(20):6451–61. doi: 10.1039/d5na00616c (PMC12421429; doi:10.1039/d5na00616c)
Supplement: NA-007-D5NA00616C-s009 [file NA-007-D5NA00616C-s009.pdf]

Supplementary Information (SI) for Nanoscale Advances.
This journal is © The Royal Society of Chemistry 2025

Supplementary Information

Modulating properties of solid carbon nanospheres via ion implantation with hetero-ions

Joyce B. Matsoso[1,2], Kamalakannan Ranganathan[3,4], Daniel Wamwangi[4,5], Rudolph Erasmus[4,5], Neil J. Coville[1,4]*, Trevor Derry[4,5]*

[1]Molecular Sciences Institute, School of Chemistry, University of the Witwatersrand, Johannesburg, 2050, South Africa

[2]Department of Inorganic Chemistry, University of Chemistry and Technology Prague, Technická 5, 166 28, Prague 6, Czech Republic#

[3]Department of Physics, Sona College of Technology, Salem-636005, Tamil nadu, India #

[4]DSI-NRF Centre of Excellence in Strong Materials, University of the Witwatersrand, Johannesburg, 2050, South Africa

[5]Materials Physics Research Institute, School of Physics, University of the Witwatersrand, Johannesburg, 2050, South Africa

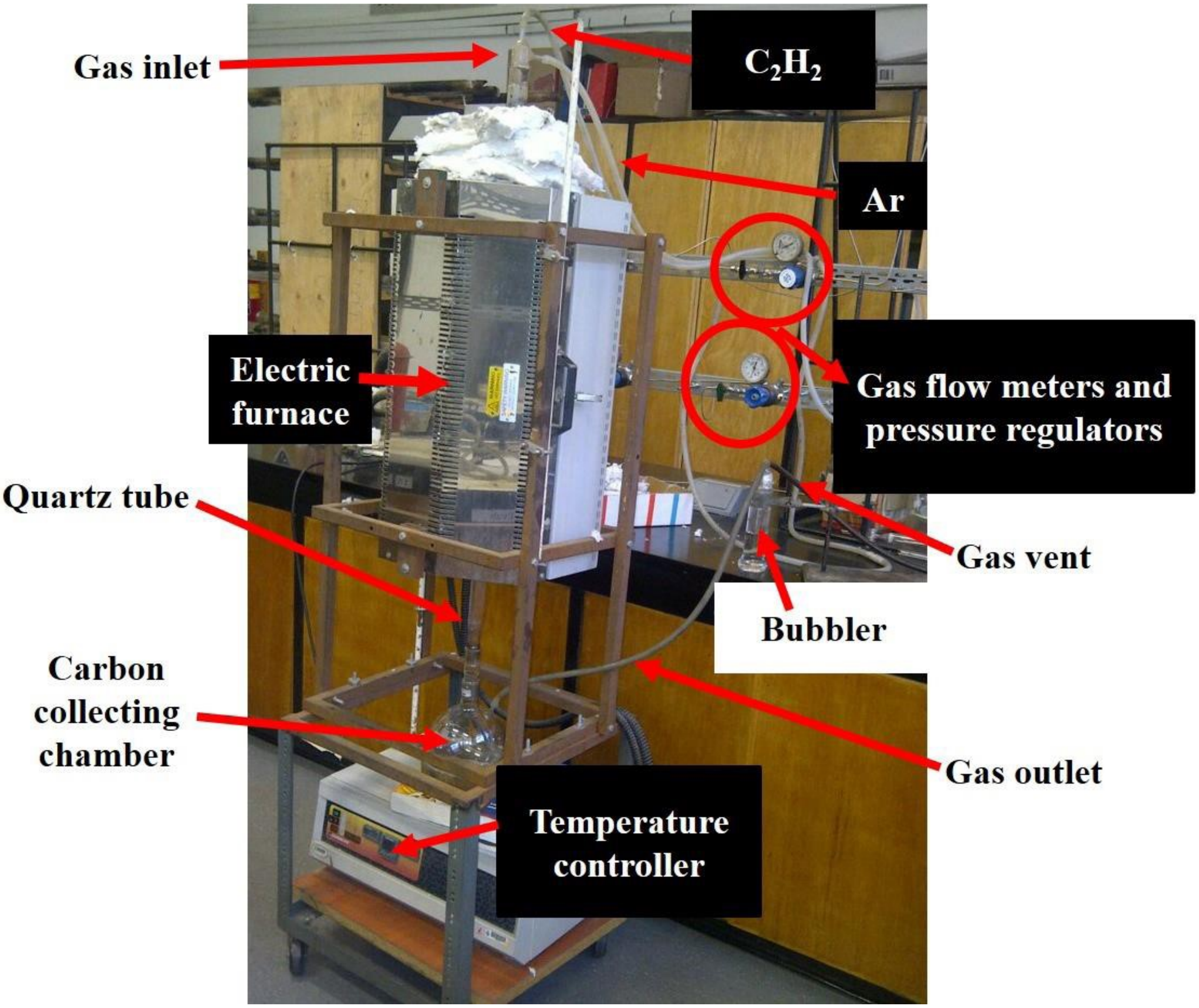

Figure S1: A vertically oriented chemical vapour deposition (CVD) reactor set up.

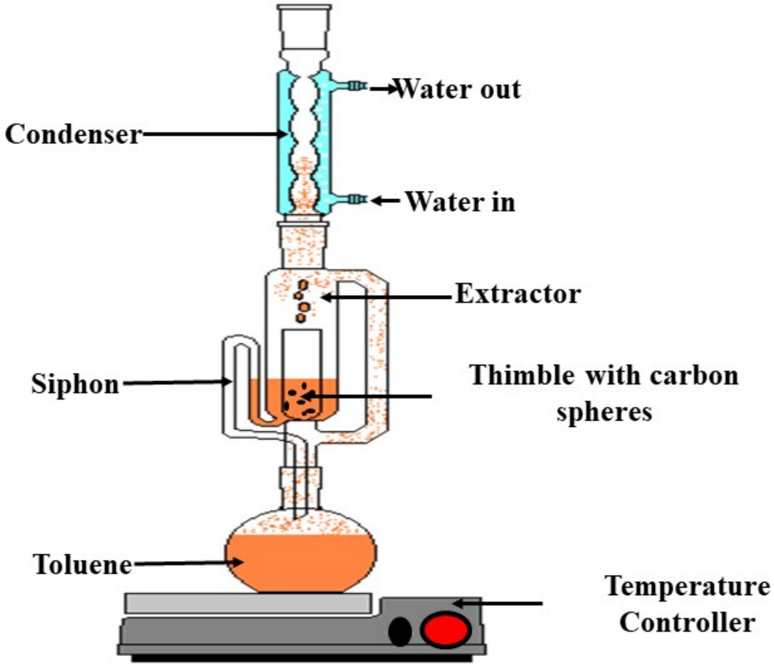

Figure S2: A conventional Soxhlet extraction set up for purification of carbon spheres[1,2].

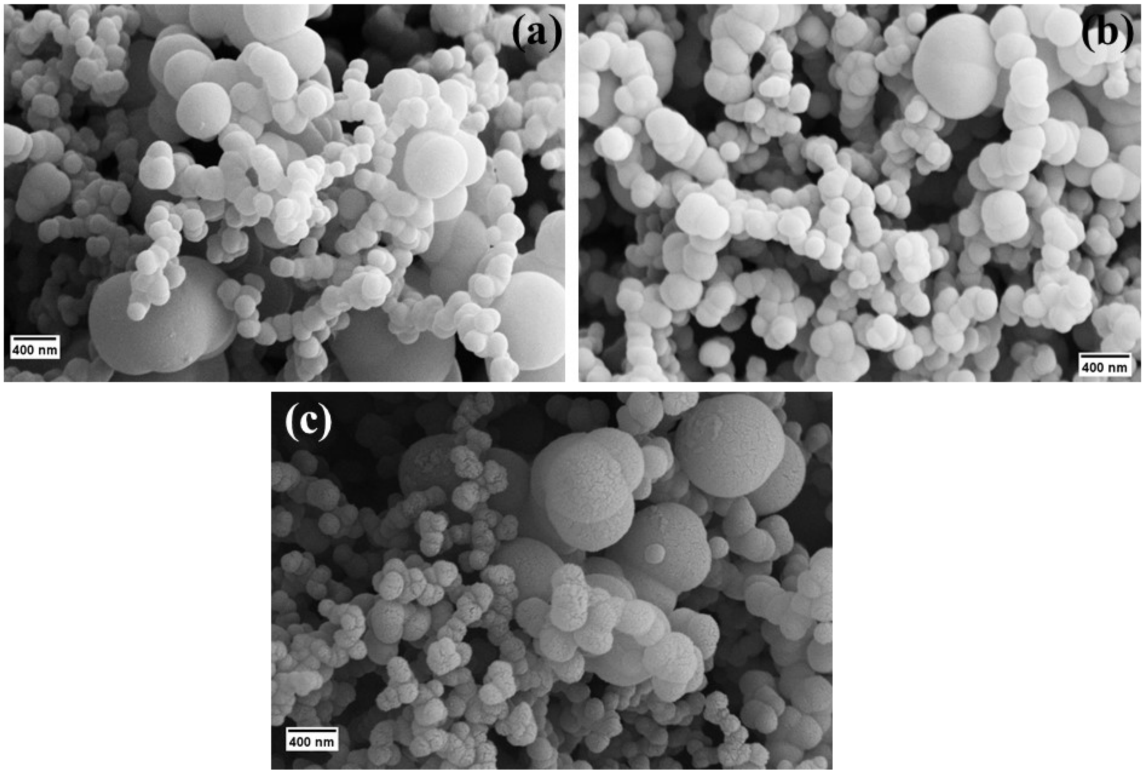

Figure S3: SEM micrographs of (a) SCS-B^+, (b) SCS-N^+, and (c) SCS-Ne^+ samples after 14 h implantation time.

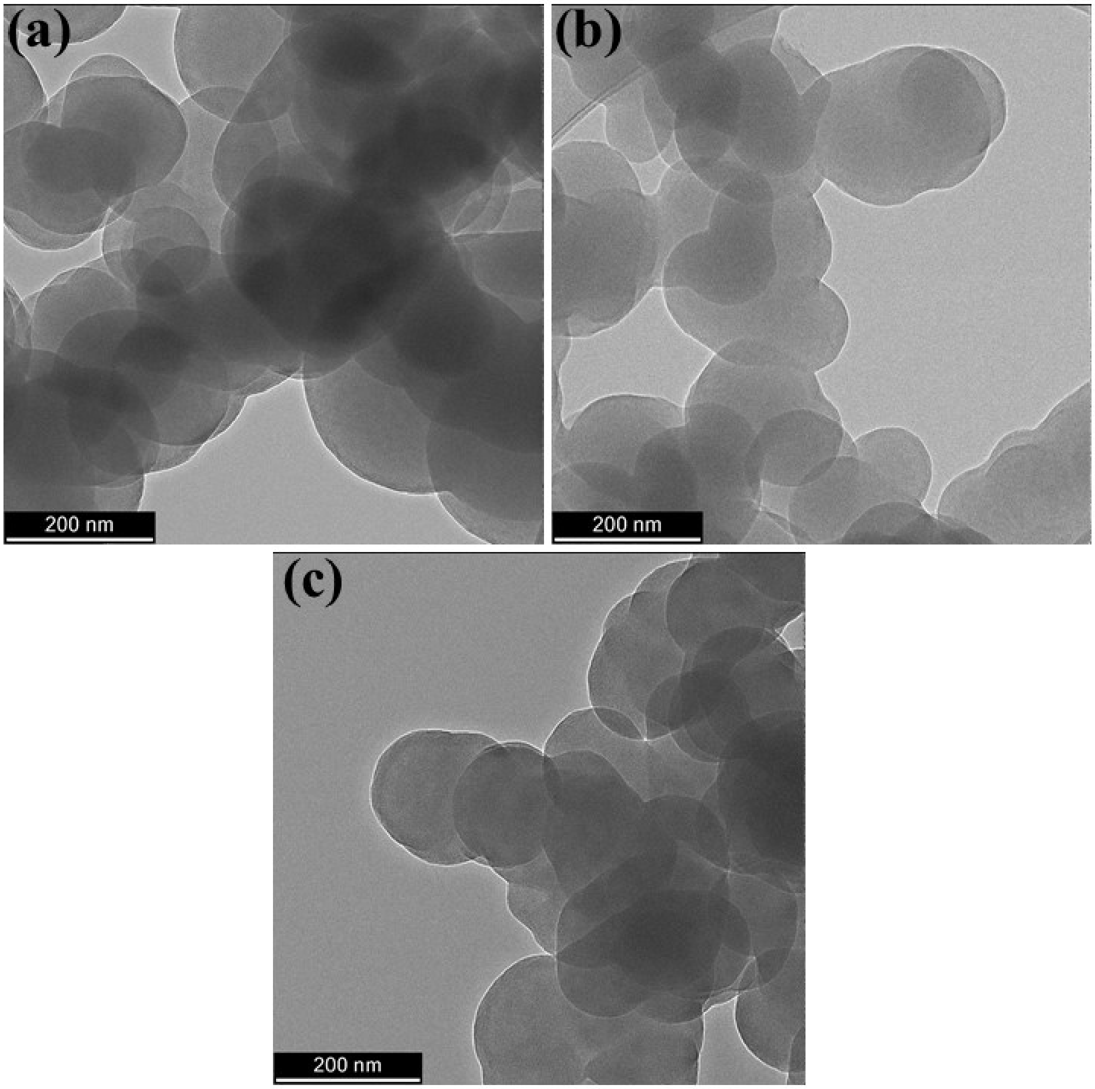

Figure S4: TEM images of (a) SCS-B^+, (b) SCS-N^+, and (c) SCS-Ne^+ samples after 14 h implantation time.

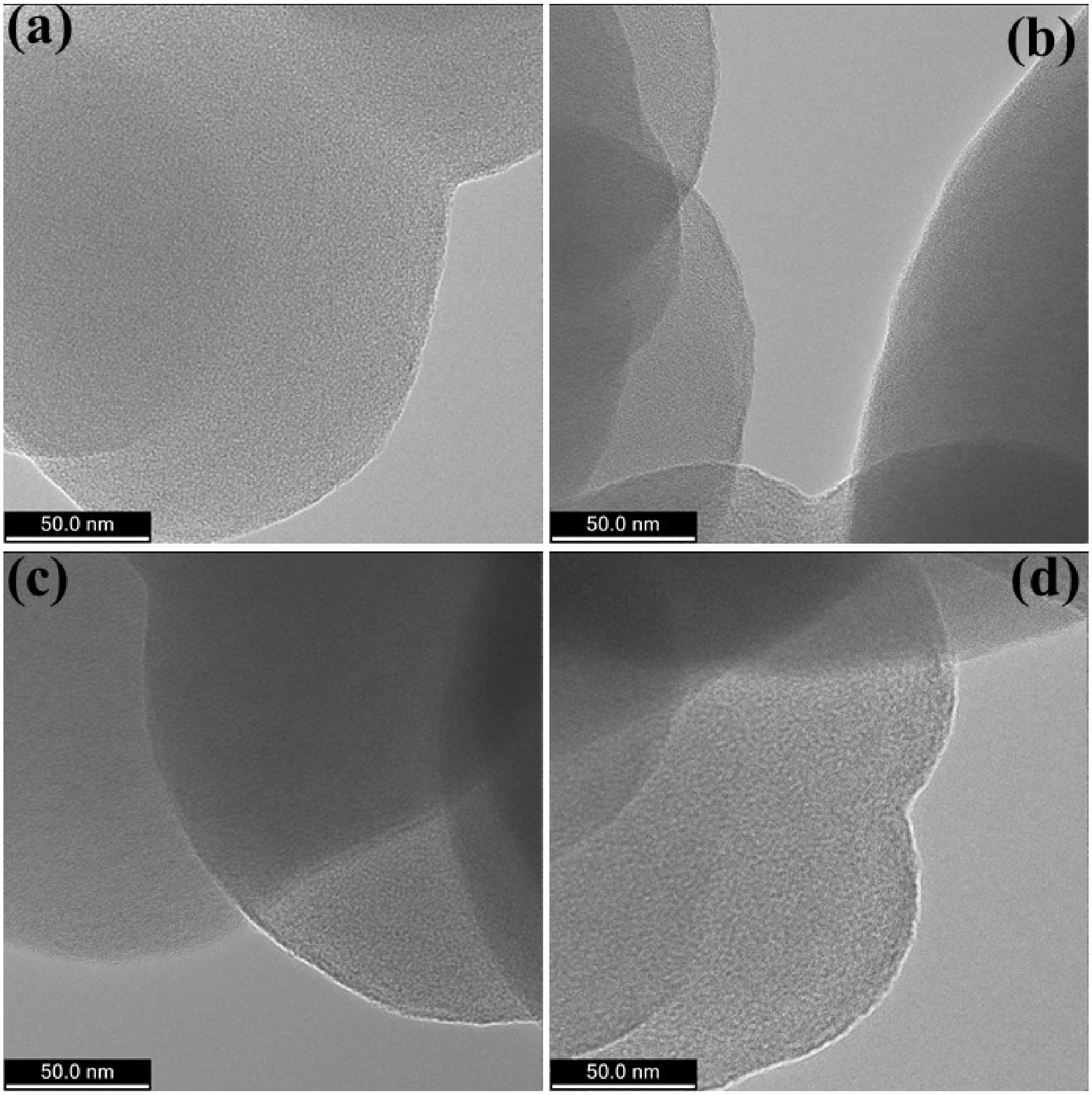

Figure S5: High magnification TEM images of (a) pristine SCS, (b) SCS-B^+, (c) SCS-N^+, and (d) SCS-Ne^+ samples after 7 h implantation time.

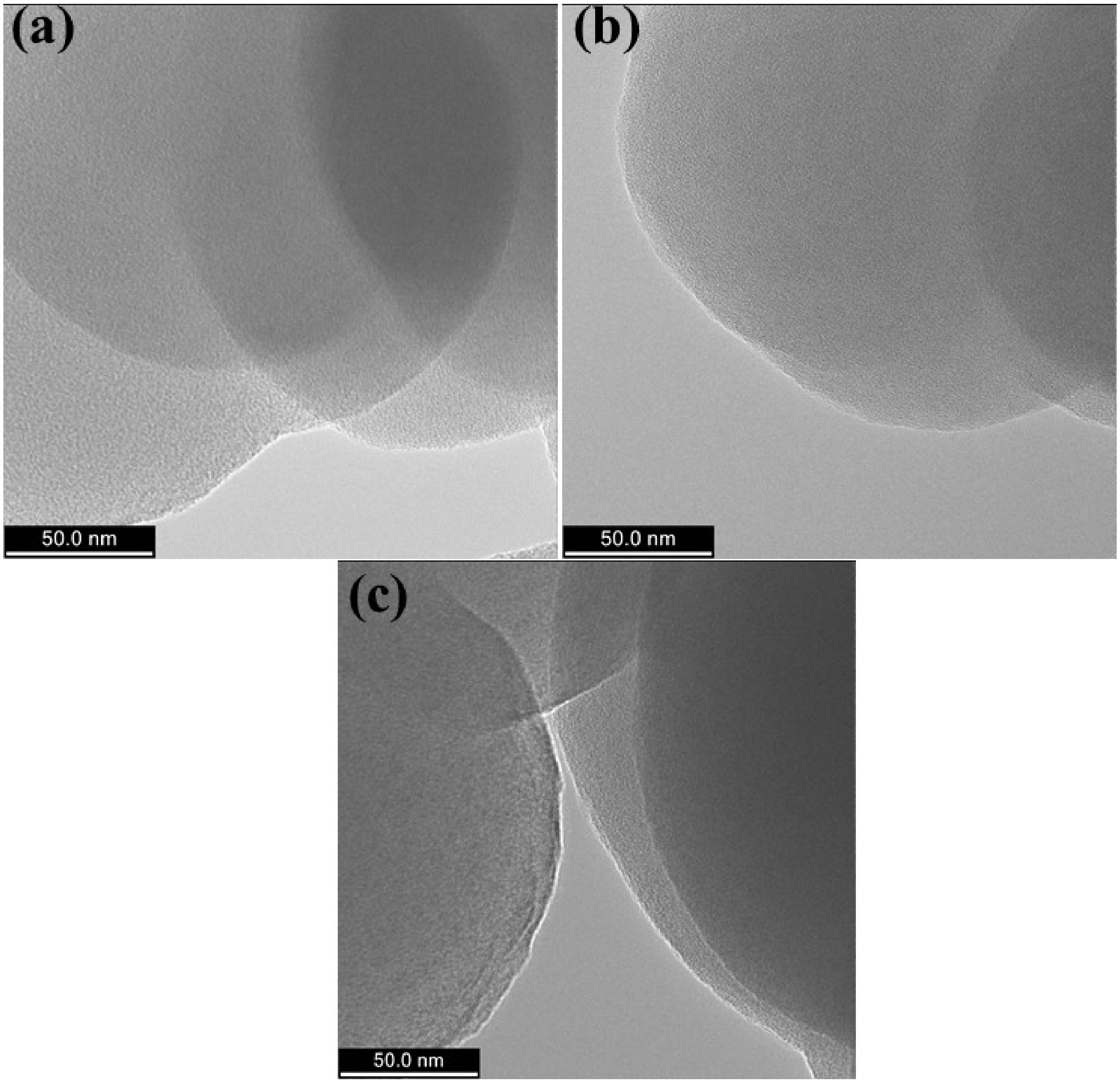

Figure S6: High magnification TEM images of (a) SCS-B^+, (b) SCS-N^+, and (c) SCS-Ne^+ samples after 14 h implantation time.

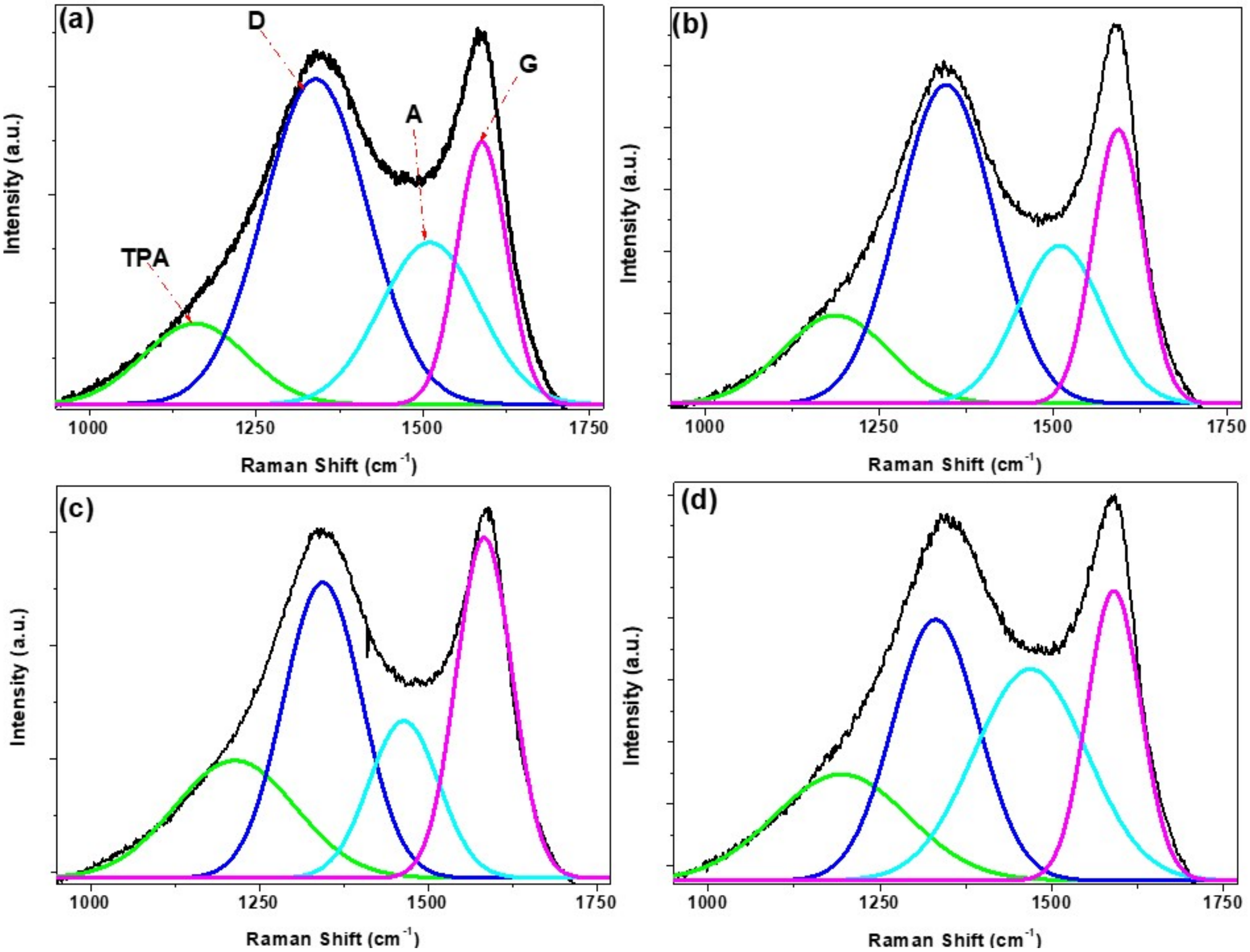

Figure S7: Peak fitted Raman spectra of (a) SCSp, (b) SCS-B^+, (c) SCS-N^+ and (d) SCS-Ne^+ samples after 14 h implantation times.

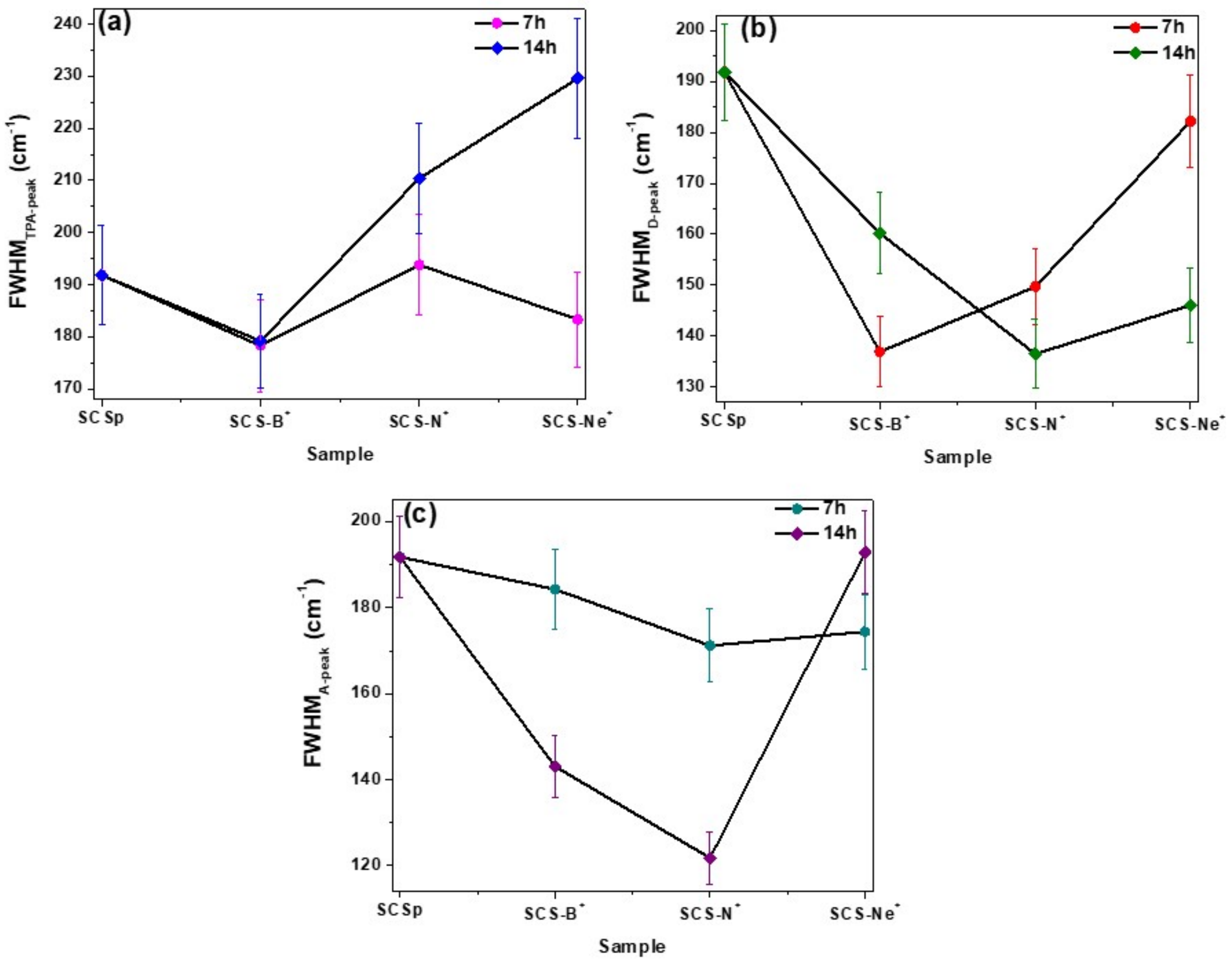

Figure S8: FWHM of the (a) TPA-band, (b) D-band, and (c) A-peak after 7 h and 14 h implantation times. The points are only linked as a guide to the eye.

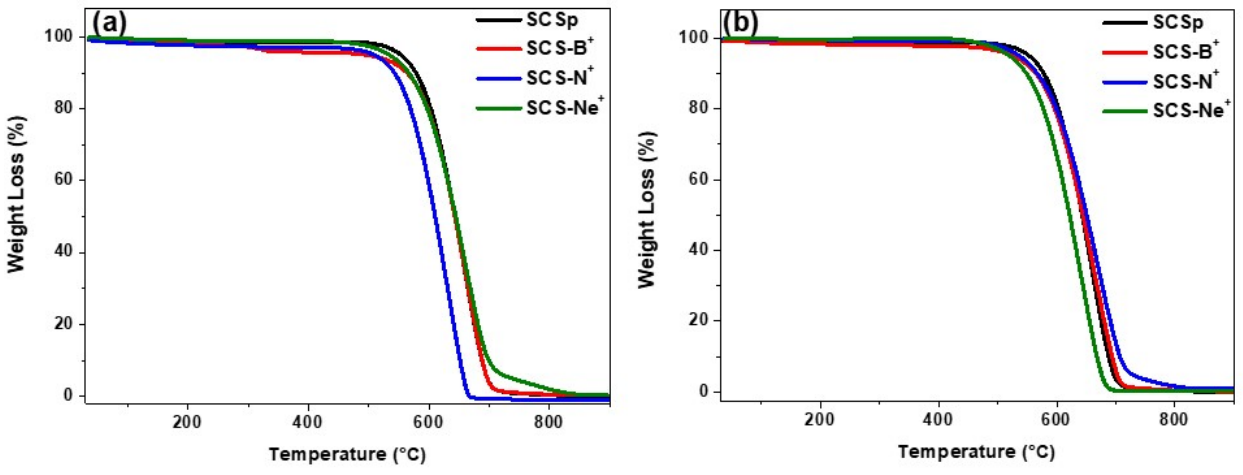

Figure S9: Thermal gravimetric curves of the SCSs samples after (a) 7 h and (b) 14 h implantation times.

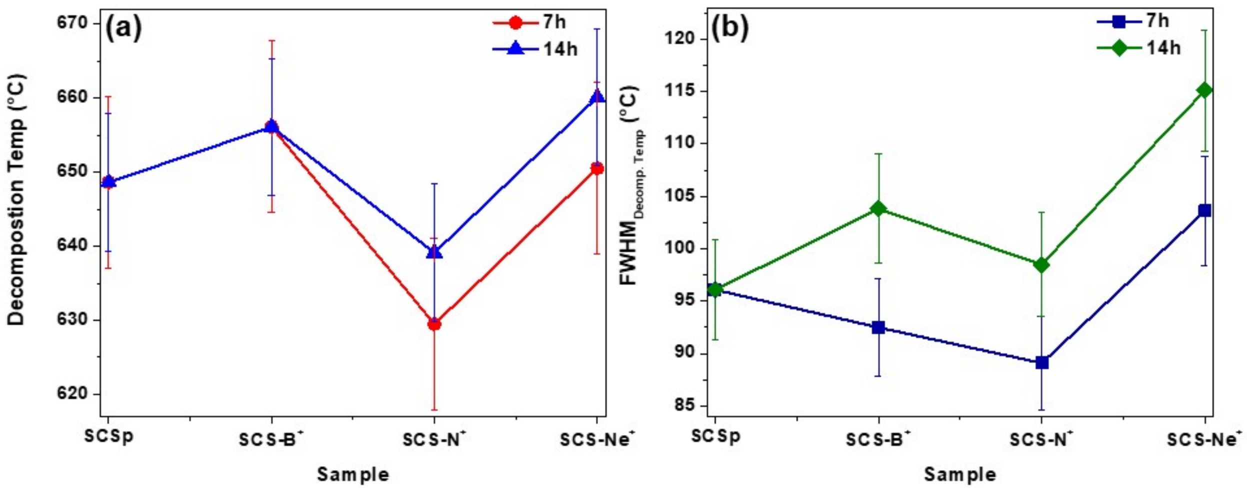

Figure S10: (a) Decomposition temperature and (b) FWHM of the decomposition temperatures after 7h and 14h implantation times. The points are only linked as a guide to the eye.

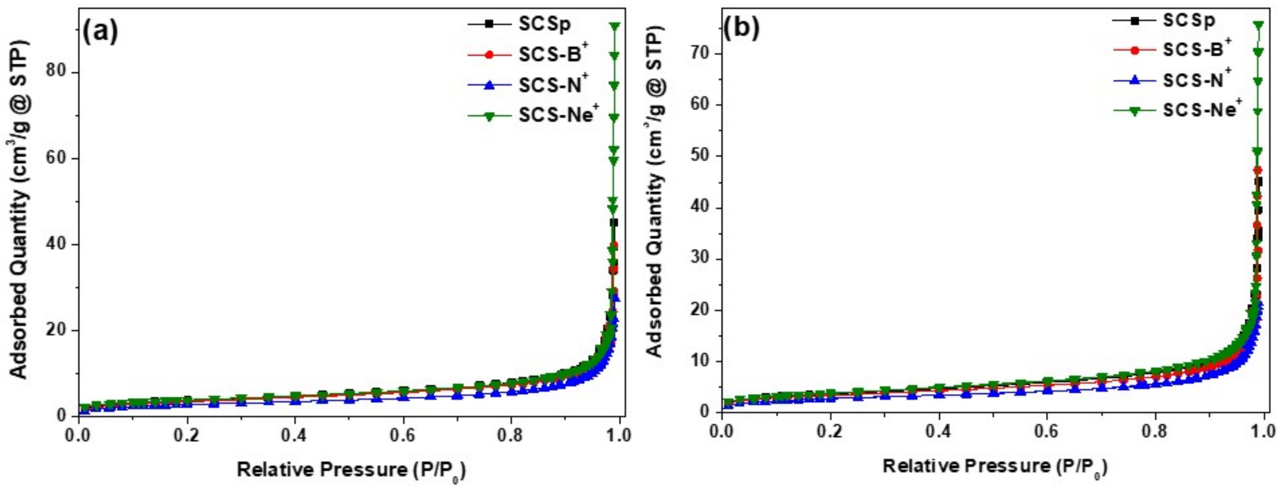

Figure S11: N_2-adsorption/desorption isotherms of the samples after (a) 7 h and (b) 14 h implantation times.

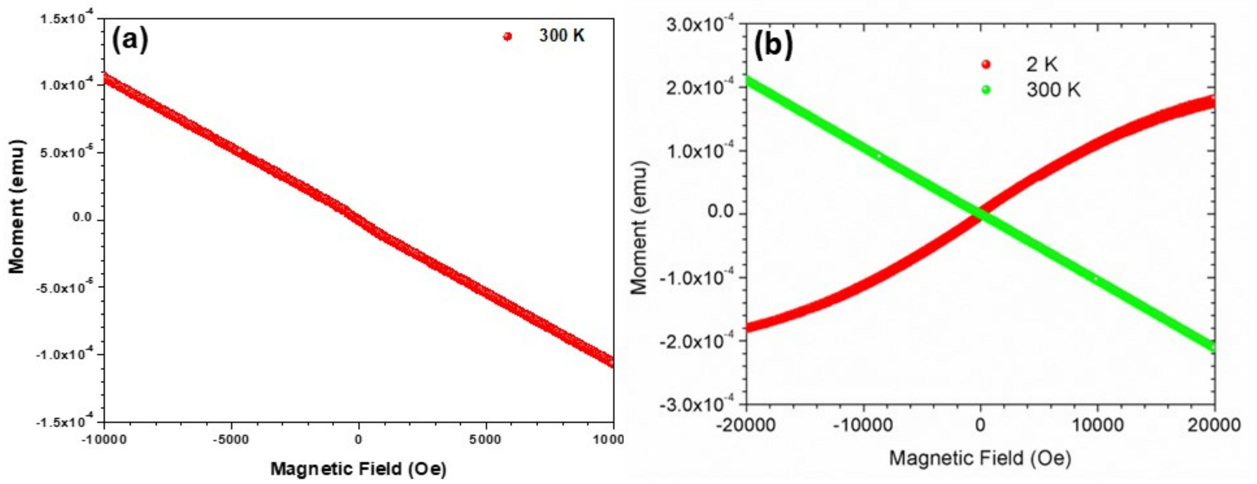

Figure S12: Isotherm curves of the SCS-N^+ samples at different temperatures after (a) 7 h and (b) 14 h implantation times.

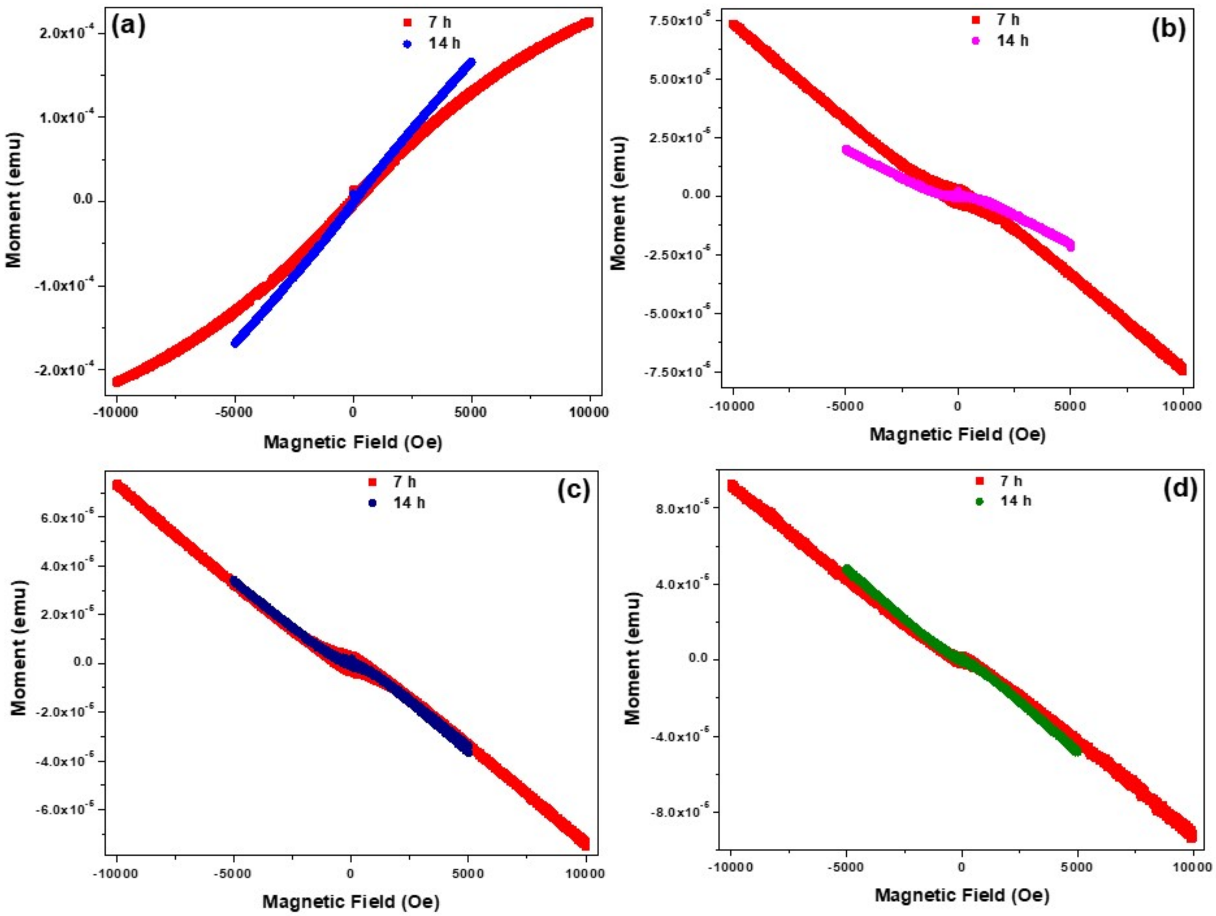

Figure S13: Isotherm curves of the SCS-B^+ samples at different temperatures after 7 h and 14 h implantation times: (a) 2K, (b) 20K, (c) 40K, and (d) 100K.

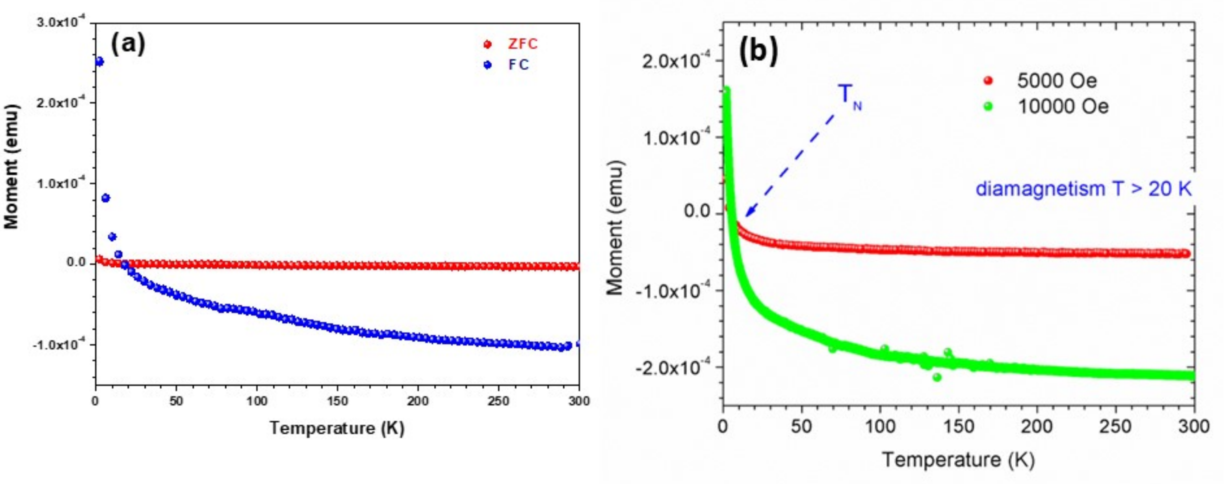

Figure S14: Isofield curves vs temperature of the SCS-N^+ samples at different field strengths after (a) 7 h and (b) 14 h implantation times.

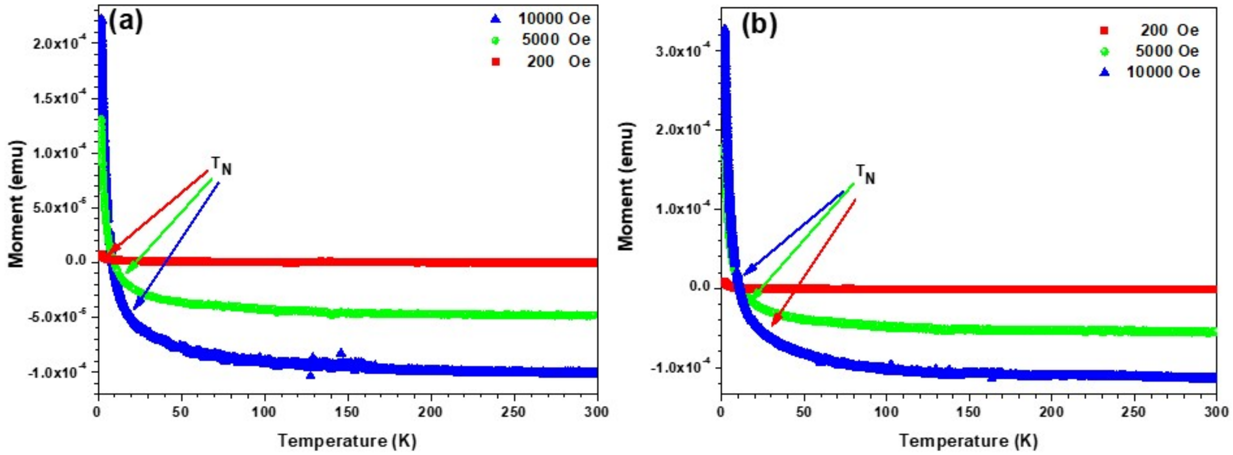

Figure S15: Isofield curves vs temperature of the SCS-B^+ samples at different field strengths after (a) 7 h and (b) 14 h implantation times.
